# Inositol 1,4,5-trisphosphate 3-kinase A overexpressed in mouse forebrain modulates synaptic transmission and mGluR-LTD of CA1 pyramidal neurons

**DOI:** 10.1371/journal.pone.0193859

**Published:** 2018-04-04

**Authors:** Byungil Choi, Hyun Woo Lee, Seojung Mo, Jin Yong Kim, Hyun Wook Kim, Im Joo Rhyu, Eunhwa Hong, Yeon Kyung Lee, June-Seek Choi, Chong-Hyun Kim, Hyun Kim

**Affiliations:** 1 Department of Anatomy, College of Medicine, Korea University, Brain Korea, Seoul, Korea; 2 Department of Psychology, Korea University, Seoul, Korea; 3 Center for Neuroscience, Brain Science Institute, Korea Institute of Science and Technology and Neuroscience Program, Division of Bio-Medical Science and Technology, KIST School, Korea University of Science and Technology, Seoul, Korea; University Paris Diderot, FRANCE

## Abstract

Inositol 1,4,5-trisphosphate 3-kinase A (IP_3_K-A) regulates the level of the inositol polyphosphates, inositol trisphosphate (IP_3_) and inositol tetrakisphosphate to modulate cellular signaling and intracellular calcium homeostasis in the central nervous system. IP_3_K-A binds to F-actin in an activity-dependent manner and accumulates in dendritic spines, where it is involved in the regulation of synaptic plasticity. IP_3_K-A knockout mice exhibit deficits in some forms of hippocampus-dependent learning and synaptic plasticity, such as long-term potentiation in the dentate gyrus synapses of the hippocampus. In the present study, to further elucidate the role of IP_3_K-A in the brain, we developed a transgenic (Tg) mouse line in which IP_3_K-A is conditionally overexpressed approximately 3-fold in the excitatory neurons of forebrain regions, including the hippocampus. The Tg mice showed an increase in both presynaptic release probability of evoked responses, along with bigger synaptic vesicle pools, and miniature excitatory postsynaptic current amplitude, although the spine density or the expression levels of the postsynaptic density-related proteins NR2B, synaptotagmin 1, and PSD-95 were not affected. Hippocampal-dependent learning and memory tasks, including novel object recognition and radial arm maze tasks, were partially impaired in Tg mice. Furthermore, (R,S)-3,5-dihydroxyphenylglycine-induced metabotropic glutamate receptor long-term depression was inhibited in Tg mice and this inhibition was dependent on protein kinase C but not on the IP_3_ receptor. Long-term potentiation and depression dependent on N-methyl-d-aspartate receptor were marginally affected in Tg mice. In summary, this study shows that overexpressed IP_3_K-A plays a role in some forms of hippocampus-dependent learning and memory tasks as well as in synaptic transmission and plasticity by regulating both presynaptic and postsynaptic functions.

## Introduction

Inositol 1,4,5-trisphosphate 3-kinase A (IP_3_K-A) has emerged as an important molecule for synaptic plasticity owing to its abilities to convert inositol trisphosphate (IP_3_) to inositol tetrakisphosphate (IP_4_) as well as to bind to F-actin and microtubules [1–6]. Although IP_3_K-A is thought to be an intracellular calcium (Ca^2+^) signaling regulator through the IP_3_ receptor (IP_3_R) pathway [1, 2], IP_3_K-A deletion in knockout (KO) mice does not change the net content of IP_3_ [7] but results in an increase in IP_3_ turnover in hippocampal synaptosomes [8]. The increased IP_3_ metabolism in the absence of IP_3_K-A appears to be due to improved inositol polyphosphate 5-phosphatase activity, which maintains the basal IP_3_-mediated Ca^2+^ release in the endoplasmic reticulum (ER) [8].

Previous studies showed that IP_3_K-A migrate toward F-actin-enriched dendritic spines via the N-terminal actin-binding domain [5] and that IP_3_K-A binds directly to activated Rac1 to modulate actin dynamics in dendritic spines [3]. Thus, overexpression of IP_3_K-A in cultured hippocampal neurons promotes dendritic spine formation through Rac1 interaction and actin remodeling. The N-terminus of IP_3_K-A can further interact with dendritic microtubules in an activity-dependent manner, and this interaction is inhibited by protein kinase A-dependent phosphorylation of Ser-119 within IP_3_K-A [4]. The IP_3_K-A kinase activity, however, is not essential for either the actin-binding [5] or spine-forming activities of IP_3_K-A [3]. Thus, IP_3_K-A exerts many physiological effects, including kinase activity-dependent Ca^2+^ homeostasis and kinase activity-independent neuronal cytoskeletal remodeling.

One way IP_3_K-A modulates neuronal function is by changing expression following neuronal activity. Hippocampal learning, such as water maze training, significantly enhances the expression of IP_3_K-A in the stratum radiatum of the CA1 and the molecular layer of the inferior blade of the dentate gyrus (DG) [9]. However, prolonged neuronal excitation by kainic acid treatment depletes the expression of IP_3_K-A in cerebral cortical neurons [10]. These data suggest that the expression of IP_3_K-A is regulated by neuronal activity in a cell type-dependent manner. Moreover, changes in IP_3_K-A expression levels affect some synaptic and cognitive functions. For example, IP_3_K-A KO mice show a loss of long-term potentiation (LTP) in granule cell synapses of the DG and a deficit in hippocampus-dependent learning tasks, such as novel object recognition [3], whereas CA1 pyramidal neuron synapses show an increase in LTP [7]. Recently, we demonstrated that IP_3_K-A KO mice also have a high level of innate fear and anxiety to aversive stimuli, suggesting a role for amygdala IP_3_K-A in associating environment with emotion [11].

In the present study, to further clarify the activity-dependent role of IP_3_K-A in brain function, we used a gain of function approach, generating a mouse line that conditionally overexpresses IP_3_K-A in the forebrain region of the mouse brain. Thus, we developed a tetracycline-inducible transgenic (Tg) mouse line that enables the transient overexpression of IP_3_K-A only in the excitatory neurons of the forebrain. Using these Tg mice, we demonstrated here that the inducible overexpression of IP_3_K-A *in vivo* impaired the performance of spatial memory tasks and blocked metabotropic glutamate receptor (mGluR)-dependent long-term depression (LTD) expression of CA1 pyramidal neuron synapses. The properties of basal synaptic transmission and synaptic plasticity were changed presynaptically as well as postsynaptically, revealing for the first time a presynaptic role of IP_3_K-A *in vivo*.

## Materials and methods

### Generation and maintenance of transgenic mouse

To generate the transgenic construct for TRE- IP_3_K-A Tg mouse, mouse IP_3_K-A cDNA was obtained by PCR amplification with plasmid templates using primers containing XbaI site at both end of primers. Forward primer: 5’-CAC CAT CTA GAA CCA TGA CCC TGC CCG GGC GCC C-3’; reverse primer: 5’-AAA AGT CTA GAG CCT CAT CTC TCA GCC AG-3’. PCR fragment was cloned into the XbaI site of placZ-GFP.gcg vector and the lacZ fragment was replaced with IP_3_K-A cDNA. The final 5.6Kb construct fragment was excised from the vector with AseI digestion, isolated from the vector backbone, purified by electro-elution, and microinjected into pronuclei of fertilized mouse eggs. Analysis of founder mice for transgene was performed by Southern blot and PCR using tail biopsies. To generate double transgenic mouse with CaMKIIα-rtTA2 and TRE-IP_3_K-A, TRE-IP_3_K-A Tg mice were crossed with CaMKIIα-rtTA2 mice (provided by Isabella M. Mansuy, [12]). Tail DNA was used for PCR genotyping with the following primers. Forward primer: 5’-TGG AAG GCG AGT CAT GGC AC-3’ and reverse primer: 5’-TCG TCA AGA GCG TCA GCA GGC AG-3’ for CaMKIIα-rtTA2 Tg mice. Forward primer: 5’-GCA GCT CGG AGC CTG AAC ACT ACT G-3’ and reverse primer: 5’-TCG TCA AGA GCG TCA GCA GGC AG-3’ for TRE- IP_3_K-A Tg mice. PCR mixtures was heated for 3 min at 94°C, followed by 35 cycles of 94°C for 30 sec, 56°C for 30 sec, and 72°C for 30 sec with a final extension of 72°C for 5 min. The PCR product of the TRE-IP_3_K-A Tg mouse was 733 bp from the transgene and 1446 bp from the mouse genome DNA that could be used for PCR internal control. To induce transgene expression, mice were dosed with 6 mg Doxycycline (Dox)/g of food. Dox-containing foods were prepared daily by mixing Dox (Sigma-Aldrich) with paste food (powdered food in sterile water, 50/50, w/v, in 2% sucrose). As WT mice, all three types of CaMKII-rtTA(−)/TRE-IP3K-A(−), -rtTA(+)/TRE-IP3K-A(−), and–rtTA(−)/TRE-IP3K-A(+) mice were used and CaMKII-rtTA(+)/TRE-IP3K-A(+) was used as Tg mice. They have all the same genetic background of c57BL/6 and all fed Dox-containing food. This study was approved by Institutional Animal Care and Use Committee of Korea Institute of Science & Technology (IACUC of KIST, 2017–004) and Korea University Institutional Animal Care and Use Committee (KUIACUC, 2016-0079-C1), and performed in accordance with the guidelines of Korea University.

### Protein isolation and Western blot analysis

Mouse tissues were isolated and homogenized in ice-cold RIPA buffer (50 mM Tris-HCl (pH 7.4), 150 mM NaCl, 1% NP-40, 0.5% sodium deoxycholate, and 1% β-mercaptoethanol) containing protease and phosphatase inhibitor cocktail (Roche Life Science). The solution was incubated on ice for 30 min, centrifuged at 15,000 × *g* for 10 min at 4°C and the supernatant was collected. Protein concentration was determined using the Bradford method (Bio-Rad). Bovine serum albumin was used as standard. Proteins were separated by electrophoresis on an 8–12% SDS-PAGE gel, transferred to a Nitrocellulose membrane (Whatman). Nonspecific binding was blocked by soaking in TBS-T buffer (50 mM Tris-HCl (pH 7.4), 150 mM NaCl, and 0.1% tween20) containing 5% skim milk for overnight at 4°C. Membranes were incubated with primary antibody for 3–6 h at room temperature (RT). After washing three times with TBS buffer, the membranes were incubated with donkey anti-mouse IgG antibody conjugated to HRP (Jackson ImmunoResearch) in TBS-T buffer containing 5% skim milk for 1 h at RT. The protein bands were visualized by using Western blot detection reagents (Thermo Scientific) and AGFA X-ray films. Quantification of band intensity was achieved by normalizing the protein level of each sample to the level of GAPDH (Santa Cruz Biotechnology).

### Subcellular fractionation of hippocampal homogenates

Biochemical fractionation was performed according to the standard methods [13]). Hippocampal tissues were homogenized in ice-cold TEVP buffer (10 mM Tris-HCl (pH 7.4), 1 mM EDTA, and 1 mM EGTA) containing 0.32 M sucrose and protease and phosphatase inhibitor cocktails (Roche Life Science). All processes were performed at 4°C or on-ice. The homogenate was centrifuged at 800 × *g* for 10 min to remove nuclei and large debris (P1). The supernatant (S1) was centrifuged at 9,200 × *g* for 15 min to obtain a crude synaptosomal fraction (P2) and a soluble cytosolic fraction (S2). P2 fraction was solubilized and centrifuged at 25,000 × *g* for 20 min to produce synaptosomal membrane (LP1) and synaptosomal cytosolic (LS1) fractions. LS1 fraction was centrifuged at 165,000 × *g* for 2 h to obtain synaptic vesicle-enriched fraction (LP2). An equal amount of fractionated protein was used for Western blot. SDS-PAGE was performed as described above.

### Immunohistochemistry

Mice were anesthetized with urethane and perfused transcardially with TBS (pH 7.4) followed by 4% paraformaldehyde (PFA) in TBS. Brains were isolated, post-fixed in 4% PFA/TBS overnight at 4°C, and cryopreserved in 30% sucrose (w/v)/TBS solution at 4°C until brain subsided. Brains were embedded into optimal cutting temperature (OCT) compounds and frozen with dry-ice. The sections were made into a 40 μm thick cryostat and incubated at RT in TBS + 0.1% Triton X-100 containing 5% normal goat serum (VECTOR Laboratories, CA). The sections were then incubated with primary antibodies diluted in TBS + 0.1% Triton X-100 overnight at 4°C, washed with TBS 3 × 10min and then incubated with goat anti-mouse IgG secondary antibodies conjugated with Cy3 (Jackson ImmunoResearch, PA) for 45 min at RT. The sections were mounted on a slide glass with CRYSTAL (Biomeda, CA) and photographed with an LSM510 confocal microscope (Zeiss, Germany). The control sections were created by omitting the primary antibody without the fluorescent signal. Primary antibodies such as mouse anti-IP_3_K-A (1:1000, [3]) and rabbit anti-PSD95 (1:500, Zymed) were used.

### Golgi staining

2-month-old mice fed Dox-containing food for 2 weeks were used for Golgi staining. Littermate mice were sacrificed by deep anesthesia and transcardial perfusion with 4% PFA. The brains were post-fixed in 4% paraformaldehyde for 24 h and then stained with a rapid Golgi protocol. Briefly, the blocks containing DG were rinsed with 0.1 N sodium cacodylate buffer and stored in 2.25% potassium dichromate and 0.4% osmium tetroxide solution for 4 days in the dark at 20°C. The blocks were then transferred to a 0.75% silver nitrate solution and maintained in a dark room at 20°C for 3 days. After the process for electron microscopy (dehydration through a graded series of ethanol and then propylene oxide, followed by embedding in Epon/Araldite), coronal sections (80 μm thick) were cut from the embedded samples using a sliding microtome. The sections were re-embedded on glass slides and allowed to cure. Fully impregnated dentate granule cells and CA1 pyramidal cells were randomly selected in each animal and observed under a light microscope (AxioSkop; Zeiss).

### EM analysis

Mice were anesthetized deeply with sodium-pentobarbital and transcardially perfused with normal saline before 2% PFA, and 2.5% glutaraldehyde in 0.1M phosphate buffer (pH 7.4). The pre-fixed brain was removed and stored in the same fixative overnight at 4°C. Hippocampus were dissected from pre-fixed brain and then washed with the same buffer several times. Tissues were post-fixed with 2% osmium tetroxide for 2 h, dehydrated through an ascending ethanol series and embedded with Epon mixture. Polymerized blocks were fine trimmed into the hippocampal region of the blocks and sections were cut to 70 nm thickness using ultramicrotome. Electron micrographs containing synapses and synaptic vesicles randomly selected in the region of interest at hippocampus were taken under transmission electron microscope at the accelerating voltage of 75 kV (Hitachi H-7650).

### Hippocampal slice preparation and electrophysiological recordings

From 10 days after the offspring was born, paste food containing Dox was given to the mother, and the transfer of Dox to the offspring was confirmed by Western blot method (S1 Fig). This administration continued until the day of the electrophysiological experiment. Hippocampal slices (300 zμm thick, male mice 3–5 weeks old) were prepared from both sides of male mouse brain. In brief, hippocampal containing region was dissected out using cold oxygenated (95% O_2_/5% CO_2_) artificial CSF (aCSF): 124 mM NaCl, 2.5 mM KCl, 26 mM NaHCO_3_, 1.25 mM NaH_2_PO_4_H_2_O, 11 mM Glucose, 0.5 mM CaCl_2_H_2_O, 5 mM MgCl_2_, pH 7.3. The slices were kept on the surface of the cell culture insert in an incubation chamber provided with humidified oxygen for continuous storage, and at least one hour after dissection, one or two slices were transferred to the recording chamber. Recording aCSF has 2.5 mM Ca^2+^ and 1.3 mM Mg^2+^, and the other components are the same as those of the dissecting aCSF. For field excitatory postsynaptic potential (fEPSP) measurement, single bipolar metal electrode (FHC, Bowdoinham, ME) was placed on one side of the CA1 dendritic region and the glass recording electrode filled with aCSF was positioned on the other side of 300–500 μm. Stimulating currents were delivered from the constant current isostimulator (SC-100, WECO) every 30 sec. To measure input-output synaptic responses, seven different stimulus strength of currents (10 to 190 pA) were applied twice and the slope of fEPSP were calculated. During this step, about 40–50% of the maximum stimulus current intensity, which showed the population spike response of fEPSP, was chosen as the sub-threshold stimulus for both basal and LFS or theta-burst stimulus protocol. LFS for LTD was given 900 pulses at 1Hz and single theta-burst protocol was composed of 10 bursts at 4 Hz where each burst consists of 5 pulses at 100 Hz. In case of four theta-burst stimulations for strong LTP, each burst was repeated at 5 min interval. fEPSP was collected with Dagan amplifier (EX-1), filtered at 2 kHz, and stored on a PC hard disk.

The miniature excitatory postsynaptic current (mEPSC) was obtained by whole-cell recording. For whole-cell recordings, healthy pyramidal neurons were selected from the CA1 pyramidal cell body layer by a 40-fold objective infrared differential interference contrast (IR-DIC) microscope (Olympus BX51W1). For α-amino-3-hydroxy-5-methylisoxazolepropionate receptor (AMPAR)-mediated mEPSC measurement, 1 μM tetrodotoxin (TTX), 100 μM DL-2-amino-5-phosphonovaleric acid (APV), 100 μM picrotoxin, and 10 μM bicuculline were added in aCSF. For mEPSC, intracellular solution has the following components: 130 mM Cs-methanesulfonate, 20 mM CsCl, 10 mM HEPES, 2 mM MgATP, 0.3 mM Na_3_GTP, 0.2 mM EGTA, pH 7.3 (about 300 mOsm). The cell was held at -68mV and recording was performed at 30°C. Traces of 500 ms were collected at 3 sec intervals, filtered at 2 kHz, and amplified with Axopatch-1D patch clamp (Axon Instruments, USA). Cells with a series resistance greater than 15 MΩ were discarded. Most of the chemicals were purchased from Sigma and DHPG, TTX, Rö-31-8220 mesylate, calphostin C, 2-APB, KN62, KN93, and KN92 were from Tocris.

All data acquisition and analysis, except for miniature current, were performed with custom software written in Axobasic 3.1 (Axon Instruments). Representing values are expressed as Mean ± SEM. Miniature currents were analyzed using Mini Analaysis Program (v. 5.6.7, Synaptosoft Inc).

### Behavioral tests

Behavioral experiments were initiated after 2 weeks of Dox-containing food (6 mg/g of food) in mice aged 2 to 4 months, and Dox administration continued until the end of the experiments.

#### Open field test

A square black box (40 wide × 40 deep × 27 cm high) was placed in the center of a well-lit room and surrounded by black curtains. Background noise (60 dB) was provided by the white noise generator. Animal activity was measured by an automatic video tracking system (SmarTrack^®^; Smartech, Madison, WI). Animals were transferred to the experimental room 30 min before the start of test. The mouse was placed in the open field box and its locomotor activity (total distance) was measured with an automatic tracking system for 10 min. Spatial characteristics (percentage of time spent in the center or marginal area of open field box) were also measured.

#### Object recognition test

Two objects not larger than animals and immovable by animals were placed 10 cm from the edge of square black box (40 wide × 40 deep × 27 cm high). After fully handled, the mice were exposed to test arena for 10 min one day before the object recognition sample test was performed. The object recognition test consists of two types of trials: sample trial (first day, lasting 10 min) and retention trial (second and fourth day, lasting 5 min). In the sample trial, two identical objects (A and A) were placed in the box and the mouse was placed in the center of the test arena. The total time spent exploring both objects was measured. In the retention trial after 24 h from the sample trial, one of the two identical objects in the sample trial was changed to new object (A and B) and the time spent exploring objects was measured. In the retention trial after 72 h from the sample trial, the changed object in the 24 h retention trial was changed to another object (A and C). The preference, which is the ratio of exploring time to new and old objects, was used as a measure.

#### Radial arm maze test

To test spatial working memory, the radial maze test was conducted as described previously, with slight modifications [14]. The floor of the maze was made of white Plexiglas, and the wall (17 cm high) was consisted of transparent Plexiglas. Each arm (6 × 28 cm) radiated from a circular central starting platform (20 cm in diameter). A small well (1 cm deep and 1cm in diameter) containing 0.2 ml of 10% sucrose solution was placed at the distal end of each arm as rewards. The maze was elevated 60 cm above the floor and placed in a room with a number of extramaze cues, including the experimenter. Three days before pretraining, animals were habituated to a restricted water supply and fed with 10% sucrose solution for one hour every day. Pretraining was composed of three days. On the first pretraining day, each mouse was exposed to the central starting platform and allowed to explore and to habituate to the maze. On the second pretraining day, the mouse was placed in the central platform and allowed to consume rewards scattered over the arms for 5 min. On the third pretraining day, the mouse was placed at the distal end of each arm and allowed to obtain the reward from each well. A trial was finished after the subject approached to the bait and returned to the central platform, irrespective of its consuming the solution. This trial was conducted in all eight arms for each mouse. After three days of pretraining, 14 days of maze acquisition begun. During the acquisition session, all eight arms were baited with sucrose solution. The mouse was placed on the central platform and allowed to obtain all eight rewards within 5 min. A session was terminated immediately after all eight rewards had been consumed or if 5 min had elapsed, whichever comes first. An arm choice was defined as traveling more than 10 cm from the central platform. The session was conducted once a day and data were presented as blocks of two training sessions. Spatial working memory errors were measured by the number of visits to the same arm more than once, where the reward has already been obtained = number of revisits). For each training session, the error rate was calculated from the number of revisits divided by the total number of visits to any of the arms.

### Statistical analysis

Statistical analyses were conducted using SPSS v. 12 for Windows (IBM corp., Amonk, NY, USA). For data comparing two groups and electrophysiology data, unpaired two-tailed Student’s *t*-test was performed, otherwise stated. Experiments with repeatedly measured data such as behavior tests and PPF ratio were analyzed by two-way repeated measures ANOVA (SPSS) with appropriate *post hoc* tests. α = 0.05 was used to show significance (asterisk) at *P* < 0.05. Values are expressed as Mean ± SEM.

## Results

### Generation of IP_3_K-A overexpressing Tg mice

**Fig 1:**
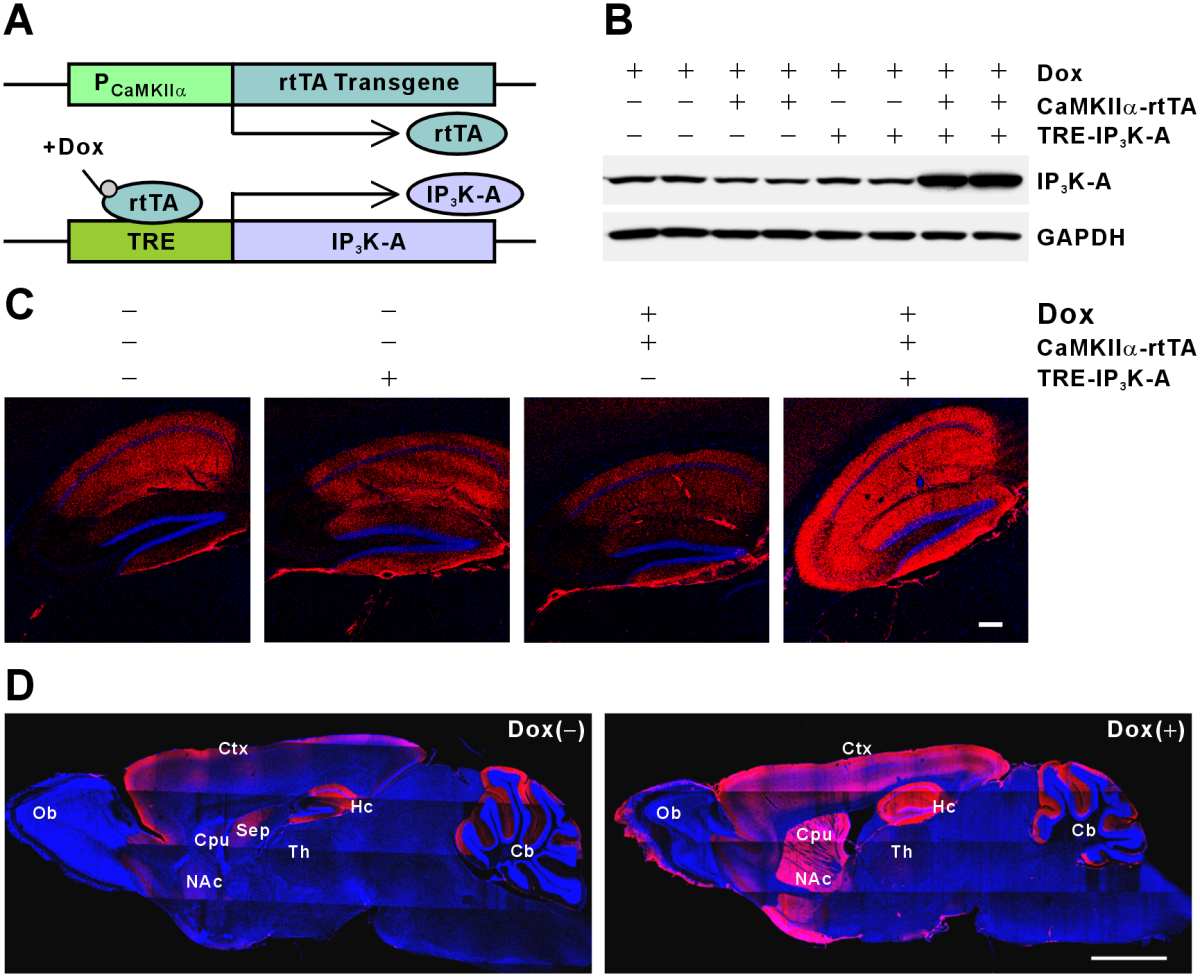
Generation of transgenic mice conditionally overexpressing IP_3_K-A. (A), Schematic diagram of the Tet-On regulatory system. Reverse tetracycline-controlled transactivator (rtTA) is expressed under the control of the tissue-specific promoter CaMKIIα. The rtTA protein binds and activates transcription of the tetracycline response element (TRE) only in the presence of Dox. (B), IP_3_K-A transgene expression in a Dox-dependent manner. IP_3_K-A expression was determined using Western blot of hippocampal lysates. (C and D), Immunohistochemical detection of IP_3_K-A protein. Coronal hippocampal sections (C) and sagittal whole brain sections (D) of WT and Tg mice fed Dox for 2 weeks immunofluorescence stained for IP_3_K-A (red) and Hoechst 33342 (blue). The induction pattern of the IP_3_K-A protein in the hippocampus was analyzed by subtracting the WT signal from the Tg signal. Scale bar, 200 μm (C) and 2 mm (D). Ctx, cortex; Hc, hippocampus; Ob, olfactory bulb; Cpu, caudate putamen; Th, thalamus; Cb, cerebellum.

The Tg mice were generated using the Tet-On system and reverse tetracycline-controlled transactivator (rtTA) protein was expressed under the control of Ca^2+^/calmodulin-dependent protein kinase II alpha (CaMKIIα) promoter activation [12]. The CaMKIIα promoter restricted rtTA protein expression to the forebrain, and the semisynthetic tetracycline, Dox, bound to rtTA and induced transcriptional activation of the IP_3_K-A gene containing the tetracycline responsive element (Fig 1A). The IP3K-A expression in the hippocampus was analyzed at Dox concentrations of 0, 2, 6 mg per gram of food. The IP3K-A expression was consistently higher in Tg mice fed Dox at 6 mg/g of food than in mice fed Dox at 2 mg/g of food (S2 Fig). The expression of IP_3_K-A in the hippocampus was increased approximately 3-fold, peaking 14 days after the mice were provided with chow containing Dox. The net induction of the IP_3_K-A protein was higher in the hippocampal CA3 region than in the CA1 region (Fig 1B and 1C). When mice were fed Dox-free chow, IP_3_K-A expression decreased slowly, returning to expression levels similar to those in wild-type (WT) mice at 6–7 weeks (S3 Fig). Enhanced expression of IP_3_K-A was detected in various regions of the Tg mouse brain, including the olfactory bulb, cortex, hippocampus, and striatum, but not in the cerebellum (Fig 1D). These results indicated that an IP_3_K-A Tg mouse line with reversible induction of IP_3_K-A in the forebrain region was successfully generated.

### Hippocampal-dependent learning and memory deficits in Tg mice

**Fig 2:**
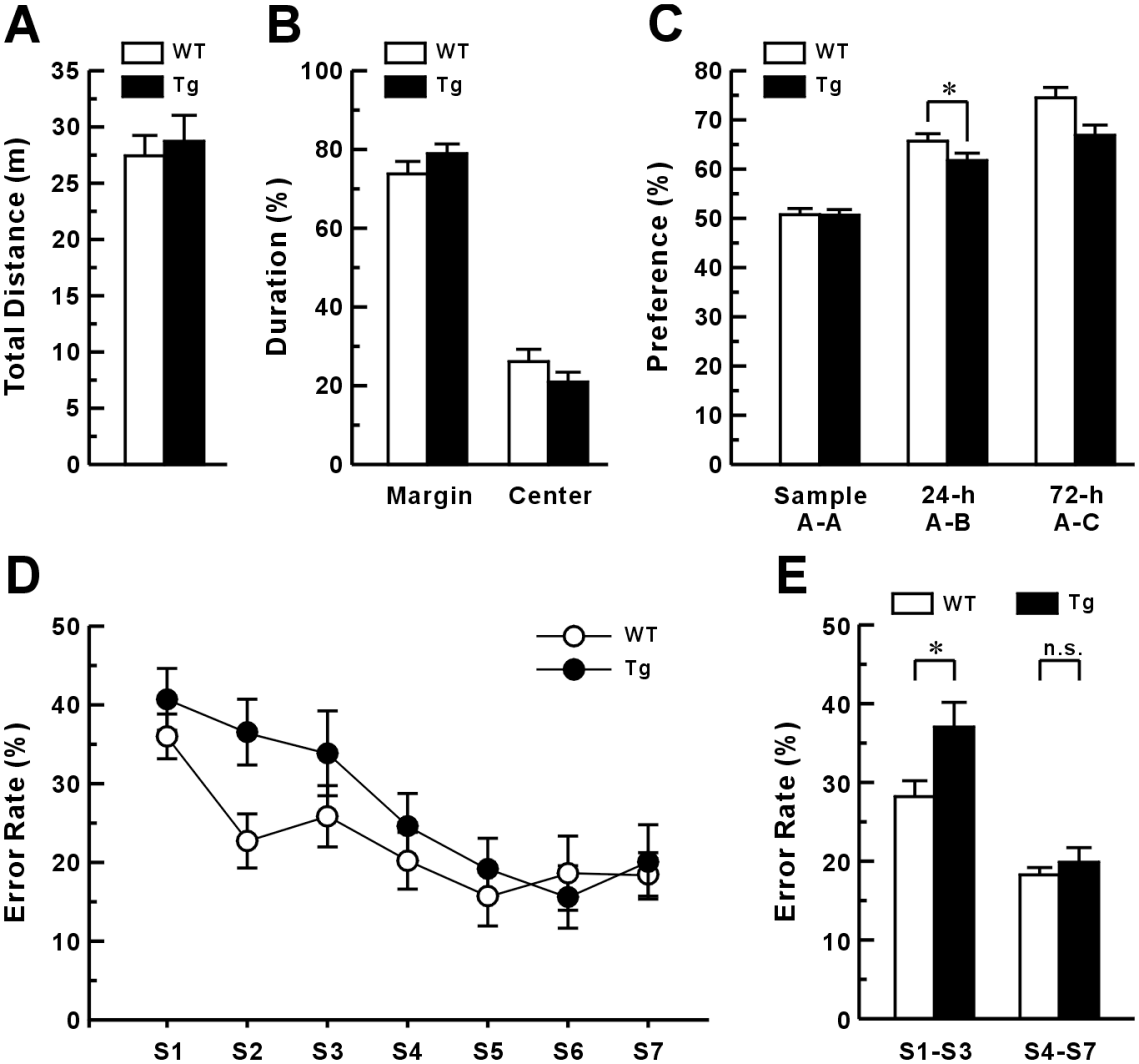
Spatial learning and memory deficits in IP_3_K-A Tg mice. In the open-field test, WT and IP_3_K-A Tg mice showed no difference in total distance traveled (A) and time spent exploring the margins or the center of the field (B) (WT, n = 11; Tg, n = 10). (C), However, IP_3_K-A Tg mice showed a mild impairment in object recognition memory. Mice were exposed to two identical or different objects (A-A, A-B, and A-C) during the sample, 24 h retention, and 72 h retention trials, respectively. The percentage of the total time that was spent investigating the novel object during each trial period was quantified as the preference percentage (WT, n = 11; Tg, n = 10). (D and E), Compared with WT mice, Tg mice showed increased error rates in the radial arm maze test. Tg mice tended to show a higher error rate in early sessions (S1–S3) (D) and showed a significant higher error rate in early phase (S1–S3), but not late phase (S4–S7) (E) (WT, n = 15; Tg, n = 12).

To gain insight into the relationship between IP_3_K-A overexpression and cognitive function, we investigated behaviors in Tg mice. First, an open-field test was conducted to assess general motor performance and anxiety levels in the Tg mice. There were no significant differences between WT and Tg mice in the total distance traveled (Fig 2A, *t*(19) = −0.444, *p* = 0.662) or percentage of time spent in the central and marginal areas of the open-field test apparatus (Fig 2B, center: *t*(19) = 1.278, margin: *t*(19) = −1.281, *p* = 0.216). These results suggested that transient IP_3_K-A overexpression does not have a significant effect on general motor activity or basal anxiety-like behavior in mice. To observe the effects of IP_3_K-A overexpression on learning and memory, we subjected Tg mice to a novel object recognition task, which is hippocampus-dependent. There was significant difference in novel objects recognition preference between groups, but the group did not affect trend of change in novel objects recognition preference (Fig 2C, condition, *F*(2,50) = 78.630, *p* < 0.05; group, *F*(1,25) = 7.148, *p* < 0.05; condition × group, *F*(2,50) = 2.797, *p* = 0.105, repeated measures ANOVA). *Post hoc* test revealed that Tg mice spent significantly less time exploring novel objects than WT mice did during the 24 h retention test; however, few effects were observed during the 72 h retention task (Fig 2C, *t*(1,25) = 6.773, *p* < 0.05, unpaired two-tailed Student’s *t*-test). We also used a radial arm maze test, another hippocampal-dependent learning task, to assess spatial learning and memory abilities of mice. Both WT and Tg mice showed a gradual decrease in the error rate, that is, re-entry into an arm already visited during the daily trial. Repeated measures ANOVA revealed that there is no difference in error rate between groups and the group did not affect the error rate trend (Fig 2D, session, *F*(6,150) = 9.255, *p* < 0.05; group, *F*(1,25) = 2.781, *p* = 0.108; session × group, *F*(6,150) = 0.999, *p* = 0.428). However, the Tg mice tended to show higher error rates than WT mice did in early sessions. Therefore, we also measured the total number of errors as a percentage of total visits to each arm and found that Tg mice showed significantly higher error rates than WT mice did during the early phase of learning session (S1–S3) (Fig 2E, *t*(25) = −2.458, *p* < 0.05, unpaired two-tailed Student’s *t*-test). Analysis of the number of visits showed similar trend as error rate results. The repeated ANOVA test showed that there was no difference in the total number of visits between groups and the group did not affect the total number of visits trend (session, *F*(6,150) = 7.862, *p* < 0.01; group, *F*(1,25) = 3.228, *p* = 0.084; session × group, *F*(6,150) = 1.259, *p* = 0.280). However, the Tg mice tended to show higher number of visits than WT mice did in early sessions. Therefore, we also analyzed differences between early sessions (S1–S3) and late sessions (S4–S7), just as error rate. Consistent with error rate results, Tg mice showed higher visits than WT mice did in the early sessions (*t*(25) = −2.620, *p* < 0.05, unpaired two-tailed Student’s *t*-test). These results suggest that that Tg mice exhibited learning retardation, but not complete impairment in hippocampal-dependent learning.

### Changes in basal properties of CA3-to-CA1 synaptic transmission in Tg mice

**Fig 3:**
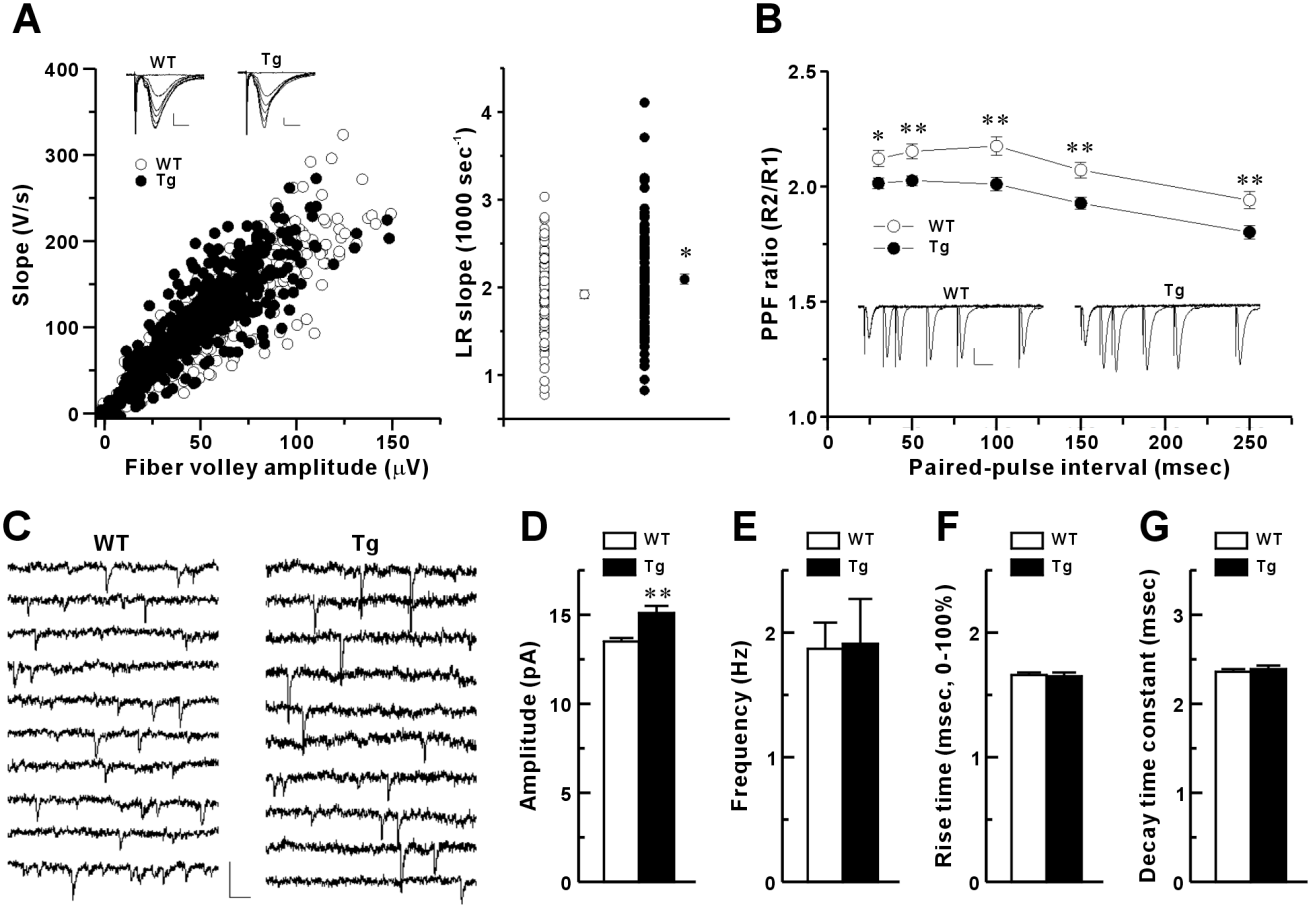
Basal physiological properties of hippocampal CA1 synapses in IP_3_K-A Tg young mice. (A), Input-output relationship of basal evoked synaptic transmission. Left, the field EPSP slopes were measured in response to six-step incremental stimulus current intensity per hippocampal slice. Scale bars: 0.5 mV, 5msec. Right, linear regression (LR) was performed on the slope values from each slice in the left I-O plot. Mean ± SEM. (WT, 1.92 ± 0.05, n = 86 slices, 19 mice; Tg, 2.10 ± 0.06, n = 97, 18; *p < 0.05). (B), Paired-pulse ratio of fEPSP responses. The ratio of the 2^nd^ fEPSP slope (R2) to the 1^st^ fEPSP slope (R1) was measured at the CA3-CA1 synapse of the hippocampal slice. WT, n = 85 slices, 19 mice; Tg, n = 94, 18; *p < 0.05 at the five intervals measured. Scale bars: 0.5 mV, 30 msec. (C-G), mEPSC properties. (C), Scale bars for all sample traces: 50 μA, 50 msec. (D), Measurements of amplitude. Mean ± SEM. (WT, 13.5 ± 0.2 pA; Tg, 15.1 ± 0.4 pA; **p < 0.001). (E), Frequency (WT, 1.87 ± 0.21 Hz; Tg, 1.91 ± 0.36 Hz; *p* = 0.92). (F), Rise time (WT, 1.66 ± 0.02 msec; Tg, 1.65 ± 0.03 msec; *p* = 0.74). (G), Decay time constant (WT, 2.36 ± 0.03 msec; Tg, 2.39 ± 0.04 msec; *p* = 0.57). The mEPSCs were measured from WT (n = 24 cells, 4 mice) and Tg mice (n = 22, 3).

To examine the basal properties of synaptic transmission in CA1 synapses, we first examined evoked synaptic responses. To measure the synaptic input-output (I-O) relationship, we analyzed the slope of the field excitatory postsynaptic potential (fEPSP) and the fiber volley amplitude generated by activating CA3 Shaffer collateral axonal fibers within the stratum radiatum area of the CA1 in hippocampal slices (Fig 3A, left plot). Each slice was activated at seven incremental input stimulus intensities and the linear regression slope values of all seven points of the fEPSP slope versus the fiber volley amplitude were plotted (Fig 3A, right plot). The mean of the linear regression slope values in Tg mice was significantly higher than that in WT mice, indicating that the CA1 synapse of Tg mouse had greater evoked synaptic transmission efficacy. This result may be due to enhanced postsynaptic functions or presynaptic properties, such as synaptic α-amino-3-hydroxy-5-methylisoxazole-4-propionic acid receptor (AMPAR) function, excitability or release probability. Next, we investigated the activity-dependent presynaptic release probability by measuring the paired-pulse facilitation (PPF) ratio of fEPSP slopes at the CA1 synapse using inter-pulse intervals of 25 to 250 msec. PPF is a type of short-term presynaptic plasticity that indirectly reflects the presynaptic release probability (Fig 3B). We observed significant difference in PPF ratio between groups and between intervals, but the group did not affect trend of change in PPF ratio (Fig 3B, interval, *F*(4,704) = 99.743, *p* < 0.01; group, *F*(1,176) = 12.149, *p* < 0.01; interval × group, *F*(4,704) = 1.382, *p* = 0.248, repeated measures ANOVA). *Post hoc* test revealed that the Tg mice showed significantly reduced PPF at all intervals, suggesting a higher activity-dependent release probability at the CA3 presynaptic terminals of Tg mice (Fig 3B, 30 msec: *t*(151.011) = 2.490, *p* < 0.05, 50 msec: *t*(144.589) = 3.236, *p* < 0.01, 100 msec: *t*(157.519) = 3.452, *p* < 0.05, 150 msec: *t*(161.517) = 3.441, *p* < 0.01, 250 msec: *t*(162.976) = 2.998, *p* < 0.01, unpaired two-tailed Student’s *t*-test). To our knowledge, this is the first report showing that IP_3_K-A expression affects activity-dependent release of presynaptic terminals. To further examine activity-independent presynaptic- and postsynaptic functions in CA1 synapses, we measured mEPSCs in the presence of tetrodotoxin, a sodium channel blocker (Fig 3C). Overexpression of IP_3_K-A led to a significant increase in the amplitude of the mEPSC (Fig 3D) but not in the frequency, rise, or decay kinetics (Fig 3E–3G) of mEPSCs, suggesting higher density or conductance of postsynaptic glutamate receptors or an increase in the quantal content of presynaptic vesicles at CA1 synapses in Tg mice. Overall, these results suggest that both presynaptic and postsynaptic properties of CA1 pyramidal neurons are altered by IP_3_K-A overexpression.

### Normal dendritic spine morphology in Tg mice

**Fig 4:**
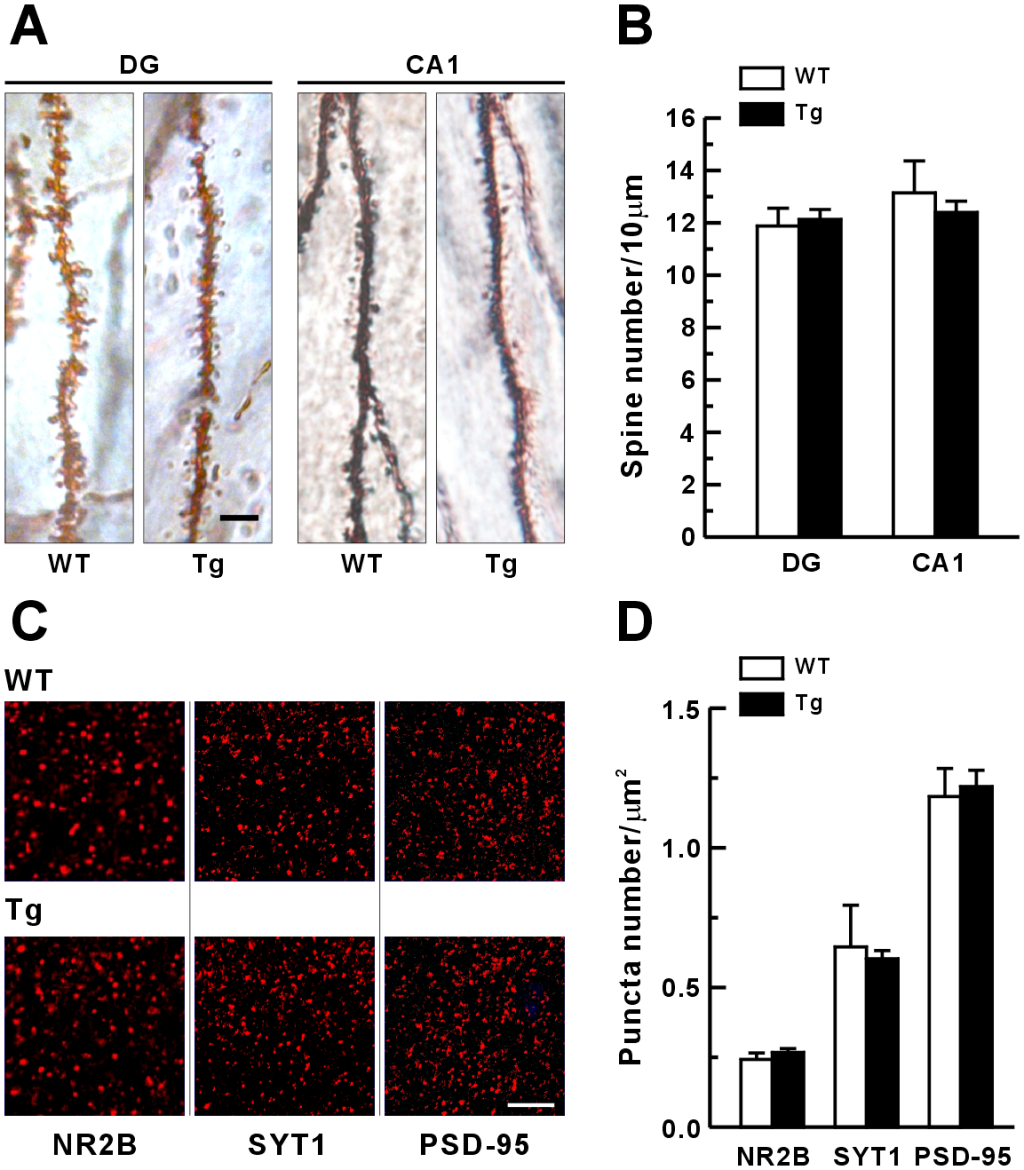
Normal density of dendritic spines and expression of synaptic molecules in IP_3_K-A Tg mice. (A), Representative images of Golgi-stained dendritic segments of CA1 and DG neurons in WT and Tg mice. Scale bar, 5 μm. (B), Quantification of dendritic spine density. Mean ± SEM. (WT, n = 60 neurons, 5 mice; Tg, n = 45, 4). (C), Representative images of immunohistochemistry of NR2B (C1), synaptotagmin 1 (SYT1; C2), and PSD-95 (C3) at the apical dendritic region of hippocampal CA1 pyramidal cells. Scale bar, 5 μm. (D), Quantification of immunofluorescent puncta of NR2B, SYT1 and PSD-95.

Given that LTP expression induces translocation of IP_3_K-A into F-actin enriched dendritic spines [4] and that IP_3_K-A-deficient mice show impaired LTP at hippocampal DG granule cell synapses [3], we investigated whether the overexpression of IP_3_K-A changed dendritic spine morphology. The dendritic spines were visualized by Golgi staining of hippocampal DG granule cells and CA1 pyramidal cells. A spine was defined as a dendritic protrusion 0.5 to 3 μm in length, with or without a head (Fig 4A and 4B). No significant difference was detected in spine density between Tg (DG granule cells, 12.13 ± 0.38 spines/10 μm; CA1 pyramidal cells, 12.4 ± 0.43 spines/10 μm) and WT mice (DG granule cells, 11.88 ± 0.68 spines/10 μm; CA1 pyramidal cells, 13.15 ± 1.22/10 μm). In addition, the expression levels of the synaptic proteins NR2B, synaptotagmin 1, and PSD-95 were not different between WT and Tg mice (Fig 4C and 4D). To determine whether any compensatory expression occurred in Tg mice, we also examined expression levels of other postsynaptic and presynaptic proteins but found none with any significant change (S5 Fig). These results indicated that overexpression of IP_3_K-A does not specifically affect the density of dendritic spines or levels of the examined synaptic proteins, suggesting that the basal IP_3_K-A expression is sufficient for homeostasis of spine morphology generation and maintenance.

### Increase in presynaptic vesicle pool size in Tg mice

**Fig 5:**
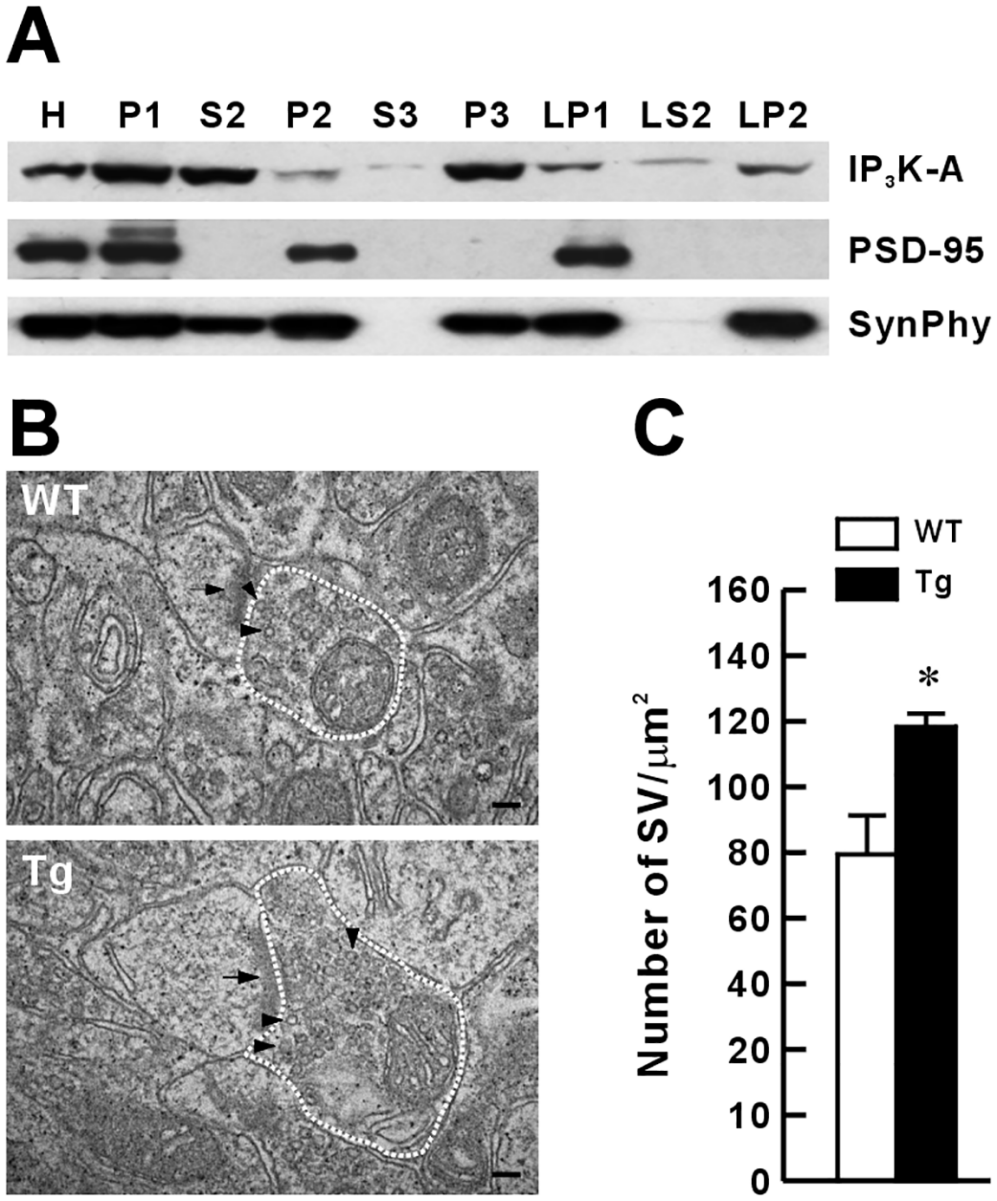
Subcellular localization of IP_3_K-A and increased number of synaptic vesicles in IP_3_K-A Tg mice. (A), Distribution pattern of IP_3_K-A in the adult mouse brain subcellular fractions. PSD-95 and synaptophysin (SynPhy) were used as controls. H, homogenates; P1, nuclei and large debris; P2, crude synaptosomes; S2, supernatant after P2 precipitation; S3, cytosol; P3, light membranes; LP1, synaptosomal membranes; LS2, synaptosomal cytosol; LP2, synaptic vesicle-enriched fraction. (B), Representative EM images of hippocampal CA1 synapses in WT and Tg mice. Scale bar: 100 mm. (C), Quantification of the number of synaptic vesicles per area (WT, n = 60 synapses, 3 mice; Tg, n = 60, 3). Presynaptic terminals with well-defined postsynaptic density (arrows) were chosen for analysis. Dashed lines indicated the presynaptic area for the analysis of synaptic vesicle (arrowheads) density per area.

Although previous studies and our data show the existence of IP_3_K-A in the postsynaptic fraction [15, 16] and in the presynaptic vesicle fraction of the mouse hippocampus (Fig 5A), we further investigated whether presynaptic changes occurred in IP_3_K-A Tg mice using electron microscopy (EM). We used EM ultrastructural analysis to determine whether IP_3_K-A overexpression affected the total pool of synaptic vesicles, which could change the release probability of neurotransmitters. The results showed that the number of synaptic vesicles in CA1 synapses of Tg mice was increased compared with that of WT mice (Fig 5B and 5C). This finding suggested that IP_3_K-A overexpression may be involved in maintaining larger synaptic vesicle pools in terminals, which would lead to a higher presynaptic release probability.

### Enhancement of the very early phase of NMDAR-dependent LTP in Tg mice

**Fig 6:**
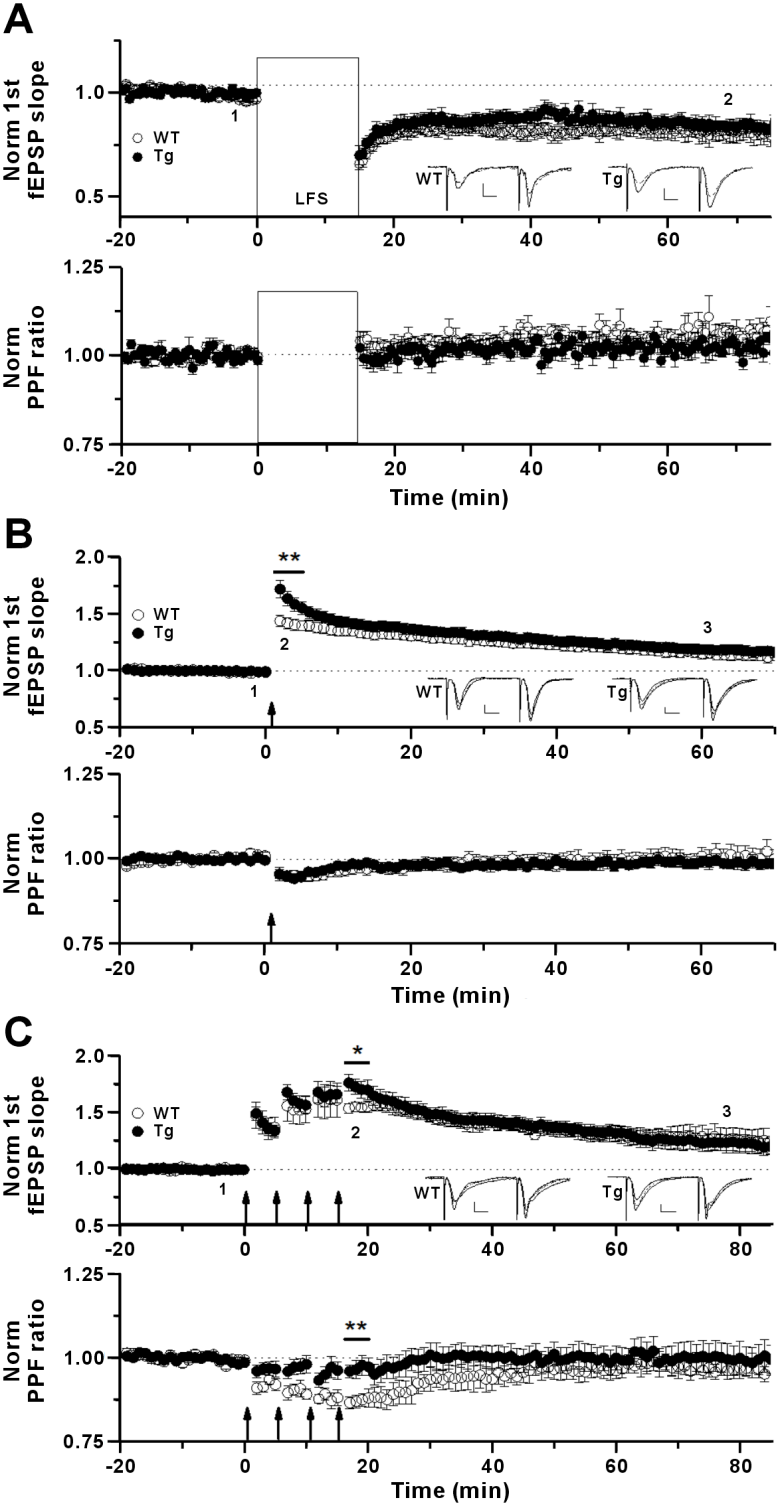
Normal fEPSP-LTD and transient increment of early LTP in young IP_3_K-A Tg mice. (A), LFS-induced LTD. Upper plot, LTD was induced by LFS protocol (1 Hz, 900 pulses) at the CA1 dendritic region of the hippocampal slice. The slope of fEPSP was 81.1 ± 1.5% (WT, n = 10 slices, 4 mice) and 84.0 ± 1.0% (Tg, n = 10, 4) at 60–65 min on the time axis (*p* = 0.11). Inlet traces are sample traces measured at baseline (1, thin line) and at 65 min (2, dashed line), respectively. Scale bars: 0.5 mV, 8 msec. Lower plot shows the normalized PPF ratio of the fEPSP slopes. PPF was measured at 60 to 65 min after induction of LTD (WT, 1.06 ± 0.80E-2; Tg, 1.02 ± 0.79E-2). (B), Single theta-burst induced LTP. Upper plot, LTP was induced with a single theta-burst protocol. Significant difference was observed immediately for 2–6 min after the theta-burst protocol (2, bar): WT, 141.5 ± 2.1%, n = 15; Tg, 160.6 ± 2.8%, n = 20; **p < 0.001. At 58–62 min (3), WT, 115.9 ± 2.0%; Tg, 118.9 ± 1.9% of baseline; *p* = 0.28. Inlet traces are sample traces indicating baseline (1, thin line), early peak (2, thick line) and late phase (3, dashed line). Scale bars for all sample traces: 0.5 mV, 8 msec. Lower plot shows the normalized PPF ratio of the fEPSP slopes. At 2–6 min, WT, 0.95 ± 0.62E-2; Tg, 0.95 ± 0.60E-2. At 58–62min, WT, 1.00 ± 0.98E-2; Tg, 0.99 ± 0.50E-2. (C), Four theta-burst induced LTP. LTP was induced with four theta-burst protocol. Significant difference was observed immediately after the theta-burst protocol (2, bar): WT, 155.0 ± 2.3%, n = 6; Tg, 169.1 ± 4.2%, n = 8; *p* = 0.027. At 78–82 min (3), WT, 125.3 ± 4.5%; Tg, 123.5 ± 3.1% of baseline; *p* = 0.74. Inlet traces are sample traces indicating baseline (1, thin line), early peak (2, thick line) and late phase (3, dashed line). Scale bars: 0.5 mV, 8 msec. Lower plot shows the normalized PPF ratio of the fEPSP slopes. At 17–20 min (2), WT, 0.87 ± 0.012; Tg, 0.94 ± 0.014; **p = 0.002. At 78–82 min (3), WT, 0.97 ± 0.011; Tg, 0.99 ± 0.021.

To explore the role of IP_3_K-A overexpression in synaptic plasticity, we induced NMDAR-dependent LTD using single-pulse low-frequency stimulation (LFS-LTD) at CA1 synapses in young WT and Tg mice (Fig 6A) while measuring the PPF ratio (40 msec interval). A similar level of LFS-LTD was induced in both WT and Tg mice without significant changes in PPF during LTD expression, suggesting that NMDAR-dependent LTD is expressed mainly postsynaptically in both types of mice. For NMDAR-dependent LTP induction, single-theta-burst (Fig 6B) and four-theta-burst (Fig 6C) stimulation protocols were used to induce protein synthesis-independent and protein synthesis-dependent processes, respectively. Compared with WT mice, Tg mice exhibited enhanced LTP only during the very early phase induced by either protocol. The PPF ratio measurements following single theta-burst stimulation indicated that Tg and WT mice showed a similar amount of enhanced presynaptic release probability during the very early phase of LTP (Fig 6B, lower panel), suggesting an additional transiently enhanced postsynaptic function in Tg mice. In four-theta-burst LTP, a more marked contribution of postsynaptic function might explain the early increase of LTP in Tg mice because the PPF change in Tg mice was much less than that in WT mice (Fig 6C, lower panel). These results suggested that overexpressed IP_3_K-A in Tg mice is involved in a higher postsynaptic contribution to the early phase expression of NMDAR-dependent LTP compared with that of LTP in WT mice while having no significant role in NMDAR-dependent LTD.

### Inhibition of mGluR-dependent DHPG-LTD and alteration in the postsynaptic contribution to LTD expression in Tg mice

**Fig 7:**
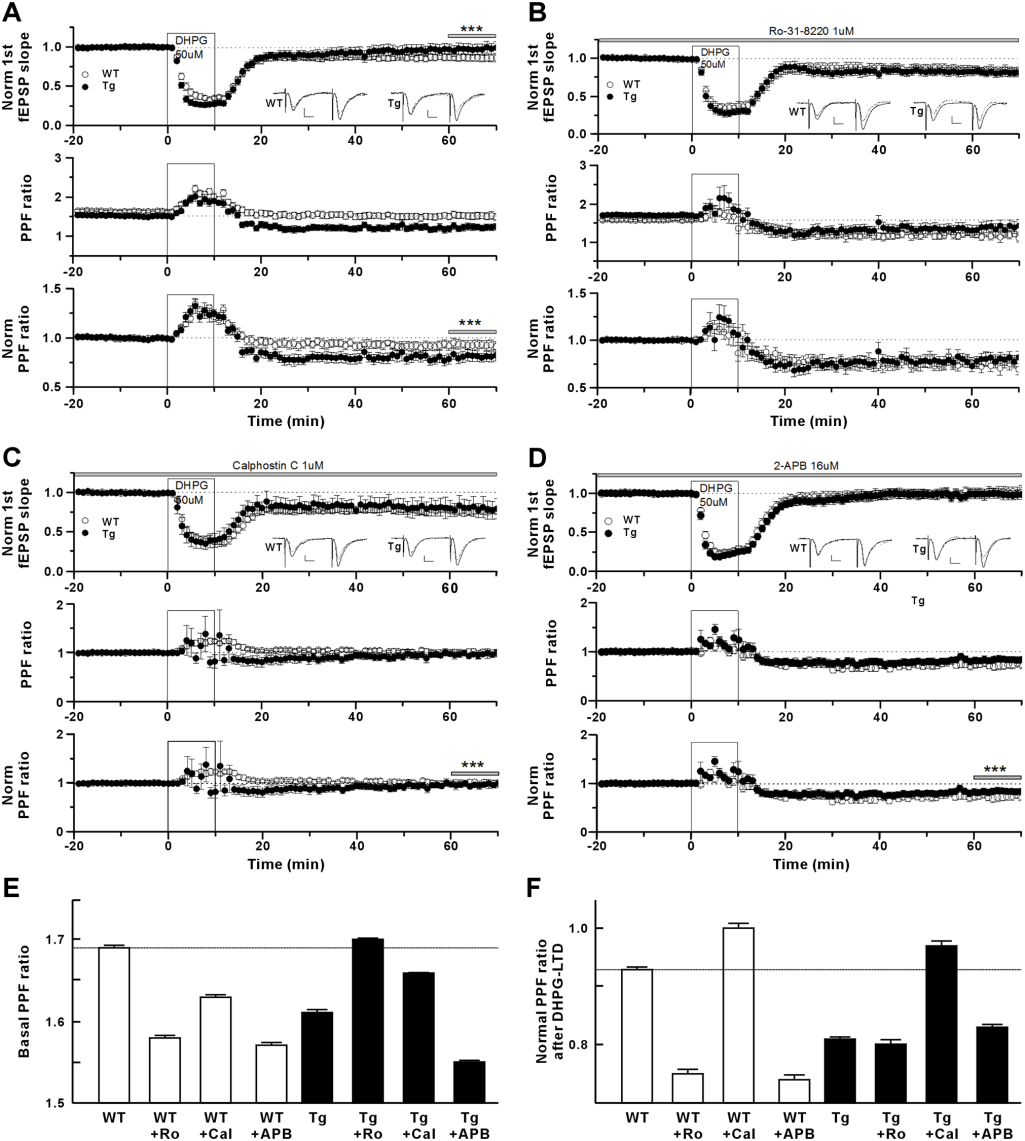
Impairment of DHPG-LTD in young IP_3_K-A Tg mice. (A), Induction and expression of DHPG-LTD. Top plot, DHPG-LTD was significantly inhibited in IP_3_K-A Tg mice compared with WT (measured at 61–70 min (grey bar); WT, 86.5 ± 0.1% of baseline, n = 20 slices; Tg, 97.7 ± 0.4%, n = 18 slices; ***p < 0.001). Middle plot, the mean PPF ratio of the baseline of Tg mice was significantly lower than that of WT (WT, 1.69 ± 0.34E-2; Tg, 1.61 ± 0.38E-2; ***p < 0.001). Bottom plot, The normalized PPF ratio after DHPG washout was significantly reduced in Tg mice compared with that of WT (measured at 61–70 min (grey bar); WT, 0.93 ± 0.20E-2; Tg, 0.81 ± 0.42E-2; ***p < 0.001). (B), DHPG-LTD in Rö-31-8220 pre-incubated slices. 1 μM Rö-31-8220 was applied throughout the entire recording period. Top plot, DHPG (50 μM) application for 10 min induced LTD in CA1 synapses in WT and Tg mice (measured at 61–70 min; WT, 83.9 ± 0.3% of baseline, n = 14; Tg, 81.4 ± 0.3%, n = 15). Middle plot, the mean PPF ratio of the baseline of Tg mice was significantly higher than that of WT (WT, 1.58 ± 0.25E-2; Tg, 1.70 ± 0.18E-2; ***p < 0.001). Bottom plot, normalized PPF ratio after DHPG washout was significantly reduced in both WT and Tg mice (measured at 61–70 min; WT, 0.75 ± 0.01; ***p < 0.001; Tg, 0.80 ± 0.01; ***p < 0.001). (C), DHPG-LTD in calphostine C pre-incubated slices. 1 μM calphostin C was applied throughout the entire recording period. Top plot, DHPG (50 μM) application induced LTD in CA1 synapses in WT and Tg mice (measured at 61–70 min; WT, 77.3 ± 0.2% of baseline, n = 6, *p* < 0.001; Tg, 80.0 ± 0.7%, n = 6, *p* < 0.001). Middle plot, themean PPF ratio of the baseline of Tg mice was similar to that of WT (WT, 1.63 ± 0.30E-2; Tg, 1.66 ± 0.02E-2; *p* < 0.001). Bottom plot, After DHPG washout, the normalized PPF ratio was almost unchanged compared to the baseline of WT and Tg mice (measured at 61–70 min; WT, 1.00 ± 0.01; Tg, 0.97 ± 0.01; *p* = 0.004). (D), DHPG-LTD in 2-APB pre-incubated slices. 16 μM 2-APB was applied throughout the entire recording period. Top plot, DHPG (50 μM) application did not induce LTD in CA1 synapses in WT and Tg mice (measured at 61–70 min; WT, 101.0 ± 0.5% of baseline, n = 18; Tg, 98.8 ± 0.3%, n = 20). Middle plot, the mean PPF ratio of the baseline of Tg mice was similar to that of WT (WT, 1.57 ± 0.40E-2; Tg, 1.55 ± 0.16E-2; *p* < 0.001). Bottom plot, the normalized PPF ratio after DHPG washout was reduced compared with that of baseline in WT and Tg mice (measured at 61–70 min; WT, 0.74 ± 0.78E-2; *p* < 0.001; Tg, 0.83 ± 0.39E-2, *p* < 0.001). Inlet traces are sample traces at scale of 0.5 mV/8 msec. All inlet traces are sample traces. Scale bars: 0.8 mV, 8 msec. (E), Summary plot of basal PPF ratio data before and after drug treatments shown in (A–D). (F), Summary plot of normalized PPF ratio data at 61–70 min period after DHPG-LTD induction shown in (A–D).

As shown in Fig 6A, LFS-LTD was normal in Tg mice; moreover, paired-pulse LFS-LTD was not affected in these mice (S6 Fig). These results suggested that NMDAR-dependent postsynaptic plasticity is relatively spared in Tg mice. Because LFS activates both NMDARs and mGluRs [17, 18], we examined mGluR-dependent LTD by applying (R,S)-3,5-dihydroxyphenylglycine (DHPG), a group I mGluR agonist [19] to hippocampal slices of young mice. The results showed that DHPG-LTD was induced in WT mice with a small decrease in the PPF ratio, suggesting a strong postsynaptic contribution to LTD expression despite some increase in the presynaptic release probability. DHPG-LTD was inhibited in Tg mice (Fig 7A, top plot). The results also indicated that, compared with WT mice, Tg mice showed a significant reduction (by approximately 5%) in the PPF of basal synaptic transmission (Fig 7A, middle plot, and Fig 7E), suggesting an increased basal presynaptic release probability, consistent with the result shown in Fig 3B. This indicated that overexpressed IP_3_K-A is involved in the enhanced presynaptic release process at the basal level. An analysis of LTD (Fig 7A, top plot) together with normalized PPF (Fig 7A, bottom plot) in Tg mice revealed a change in the postsynaptic IP_3_K-A contribution to DHPG-LTD expression. The normalized PPF ratio after DHPG washout was strongly reduced in Tg mice compared with that in WT mice, indicating enhanced presynaptic release, which might inhibit DHPG-LTD expression (Fig 7F). However, the early phase of DHPG-LTD (10–30 min after DHPG washout) was normally expressed despite the reduced PPF ratios, suggesting a strong postsynaptic contribution to the early phase of DHPG-LTD in Tg mice. During the late phase (> 40 min after DHPG washout), DHPG-LTD was blocked in Tg mice, while the PPF ratio reduction was similar to that of the early phase, indicating a decay in the postsynaptic contribution (Fig 7A, bottom plot, and Fig 7F).

In summary, these results suggested that DHPG-LTD is expressed both presynaptically and postsynaptically in WT mice, but the postsynaptic activity overcomes the presynaptic activity, resulting in LTD. In Tg mice, the early phase of DHPG-LTD is expressed by a strong postsynaptic component, which overcomes the presynaptic effect, and the late phase of DHPG-LTD is inhibited, possibly due to the decay of the postsynaptic component while the enhanced presynaptic contribution is maintained. Therefore, overexpressed IP_3_K-A appears to contribute both presynaptically and postsynaptically to DHPG-LTD, in which a change of postsynaptic IP_3_K-A contribution determines the level of DHPG expression, with an inhibitory role of overexpressed presynaptic IP_3_K-A.

### IP_3_K-A overexpression reveals differential roles of PKC in presynaptic release probability and a postsynaptic role of PKC in DHPG-LTD

Activation of the mGluR signaling pathway produces diacylglycerol (DAG) and IP_3_ from phosphatidylinositol 4,5-bisphosphate (PIP_2_) via phospholipase C (PLC) activation [20, 21]. Both DAG and IP_3_-mediated Ca^2+^ efflux from the ER activate PKC, which is involved in the presynaptic release machinery [22, 23]. Because it is unclear how these signaling molecules work with one another, we looked at the roles of the PKC and IP_3_R pathways in synaptic transmission and in DHPG-LTD of CA1 synapses. To examine the role of the PKC pathway, the PKC inhibitor Rö-31-8220 (1 μM) was applied to hippocampal slices prior to DHPG administration. The results showed that pretreatment with Rö-31-8220 significantly reduced the baseline PPF ratio by approximately 7% (Fig 7A, middle plot, and Fig 7E, WT without Rö-31-8220, 1.69 ± 0.34E-2 vs Fig 7B, middle plot, WT with Rö-31-8220, 1.58 ± 0.25E-2; *p* < 0.001), indicating that inhibiting basal PKC activity increased presynaptic release in WT mice. The effect of Rö-31-8220 on PPF in WT mice was similar to that produced by overexpressing IP_3_K-A in Tg mice in the absence of Rö-31-8220 (i.e., approximately 5% reduction in the PPF ratio, Fig 7E). However, DHPG-LTD expression was not affected by Rö-31-8220 in WT mice, although the reduction in PPF was much greater (approximately 25%; Fig 7B, bottom plot, and Fig 7F). This finding suggested that PKC inhibition in WT mice facilitates the postsynaptic contribution to DHPG-LTD to overcome the effect of enhanced presynaptic release. Our results also showed that Rö-31-8220 in Tg mice increased the baseline PPF ratio by approximately 4% (Fig 7E and 7A, middle plot, Tg without Rö-31-8220, 1.61 ± 0.38E-2 vs Fig 7B, middle plot, Tg with Rö-31-8220, 1.70 ± 0.18E-2; *p* < 0.001), indicating that inhibiting PKC in Tg mice reduced basal presynaptic release, such that basal PPF in Tg mice was now similar to that in WT mice. This finding indicated that the increased basal presynaptic release caused by overexpressed IP_3_K-A is PKC-dependent. We also found that the blockade of DHPG-LTD in Tg mice was removed by Rö-31-8220, resulting in normal LTD expression while maintaining PPF ratios reduced by approximately 20% (Fig 7B and 7F). This result suggested that the PKC inhibitor may remove the postsynaptic contribution decay in Tg mice.

To confirm the effects of PKC inhibition, calphostin C (1 μM), which blocks the DAG-binding C1 domain of PKC, was also examined. We found that calphostin C pre-application reduced the baseline PPF ratio by approximately 4% in WT mice (Fig 7E and 7A, middle plot, WT without calphostin C, 1.69 ± 0.34E-2 vs Fig 7C, middle plot, WT with calphostin C, 1.63 ± 0.30E-2; *p* < 0.001), which was consistent with the effect of Rö-31-8220. We also found that DHPG-LTD was expressed normally in the presence of calphostin C in WT mice with no change in PPF during LTD at 60–70 min (Fig 7C and 7F, 1.00 ± 0.63E-2 of baseline). This result indicated that DHPG-LTD is not affected by calphostin C and that LTD is expressed postsynatptically. Our results further showed that calphostin C increased the baseline PPF ratio in Tg mice by approximately 3% (Fig 7E and 7A, middle plot, Tg without calphostin C, 1.61 ± 0.38E-2 vs Fig 7C, middle plot, Tg with calphostin C, 1.66 ± 0.02E-2; *p* < 0.001), which is similar to the change od induced by Rö-31-8220. Calphostin C also blocked the inhibitory effect of IP_3_K-A overexpression on DHPG-LTD expression with little change in PPF (Fig 7C and 7F). The effects of calphostin C and Rö-31-8220 on DHPG-LTD were similar except for their presynaptic effects in both WT and Tg mice.

In summary, both PKC inhibitors, Rö-31-8220 and calphostin C, revealed differential roles of PKC in basal presynaptic release. Basal PKC activity inhibited release probability in WT mice but facilitated it in Tg mice. The mechanism for this altered PKC role in the release process is unknown. The effect of the inhibitors on PPF during DHPG-LTD differed, with Rö-31-8220 enhancing PPF while calphostin C did not. By contrast, both PKC inhibitors blocked the inhibitory effect of IP_3_K-A overexpression on DHPG- LTD in Tg mice, in which PKC might play a role in the decay of the postsynaptic contribution to DHPG-LTD expression. However, PKC inhibition did not affect DHPG-LTD in WT mice, consistent with previous study results [24–30]. Taken together, these results suggested that overexpression of IP_3_K-A changes the roles of PKC in both presynaptic and postsynaptic processes during basal synaptic transmission and in DHPG-LTD.

### Inhibitory role of IP_3_R signaling in presynaptic release and DHPG-LTD

To further investigate the change in the IP_3_ signaling pathway induced by PLC in DHPG-LTD, we applied 2-aminoethoxydiphenyl borate (2-APB, 16 μM), an IP_3_-sensitive Ca^2+^ release inhibitor working as an IP_3_R antagonist, before the addition of DHPG (Fig 7D). The results indicated that 2-APB treatment reduced the baseline PPF in WT mice by approximately 7% (Fig 7E and 7A, middle plot, WT without 2-APB, 1.69 ± 0.34E-2 vs Fig 7C, middle plot, WT with 2-APB, 1.57 ± 0.40E-2; *p* < 0.001), similar to the reduction induced by the PKC inhibitors. This suggested that the IP_3_R, like PKC, negatively regulates basal presynaptic release in WT mice. Our results also showed that 2-APB slowly blocked DHPG-LTD in WT mice while causing a relatively greater reduction in PPF (Fig 7D, bottom plot, and Fig 7F). Therefore, the IP_3_R antagonist-induced blockade of DHPG-LTD may be caused by enhanced presynaptic release, although a postsynaptic contribution cannot be excluded especially during the early phase of LTD. We also found that 2-APB reduced the basal PPF ratio in Tg mice by approximately 4% (Fig 7E and 7A, middle plot, Tg without 2-APB, 1.61 ± 0.38E-2 vs Fig 7C, middle plot, Tg with 2-APB, 1.55 ± 0.16E-2; *p* < 0.001), which was an effect opposite to that of the PKC inhibitors. This finding suggested that presynaptic IP_3_Rs continue to negatively modulate presynaptic release in Tg mice. Application of 2-APB also blocked DHPG-LTD expression and reduced PPF in Tg mice (Fig 7D and 7F). These results revealed a role for presynaptic IP_3_Rs in DHPG-LTD in both WT and Tg mice, although they did not exclude a postsynaptic function.

All three drugs (Rö-31-8220, calphostin C, and 2-APB) blocked changes in the fEPSP I-O plot in Tg mice (S7 Fig). The relevance of CaMKII in the DHPG-LTD process was tested using pre-application of specific CaMKII inhibitors, KN62 or KN93; however, no significant effect on the expression of DHPG-LTD was observed (S8 Fig). This finding suggested that the protocol for DHPG-LTD used here did not appear to activate the CaMKII signaling pathway.

## Discussion

Previous studies using IP_3_K-A KO mice showed that IP_3_K-A targets to F-actin-enriched dendritic spines and is involved in LTP and spatial memory [3]. The F-actin bundling activity of IP_3_K-A is much higher in immature than in mature neurons [3, 8]. In the present study, to further investigate the morphological and physiological role of IP_3_K-A in neurons, we generated Tg mice overexpressing IP_3_K-A in the brain by using a Tet-On inducible system (Fig 1). Unlike the results following IP_3_K-A overexpression in cultured hippocampal neurons, transient overexpression of IP_3_K-A in adult mice did not alter the formation of dendritic spines in hippocampal CA1 and DG neurons (Fig 2A and 2B). However, Tg mice showed impairments in spatial learning and memory (Fig 2C–2E) and in the late phase of DHPG-LTD in hippocampal CA1 synapses (Fig 7A), with a mild enhancement of the very early phase of theta-burst LTP (Fig 6B and 6C). These Tg mice further demonstrated that overexpression of IP_3_K-A resulted in an increase in the presynaptic vesicle pool size and release probability and in the inhibition of mGluR-dependent LTD. This is the first report to suggest that, along with its known postsynaptic roles in the central nervous system, IP_3_K-A modulates synaptic transmission and plasticity presynaptically.

### Transient retardation of hippocampal-dependent memory

Our results from novel object recognition and radial arm maze tests suggested that overexpression of IP_3_K-A attenuated hippocampal-dependent working memory retention transiently rather than permanently. In the novel object recognition test, there was a significant impairment in working memory retention during the 24 h, but not the 72 h, test in Tg mice. There was a higher error rate during the early rather than the late phase (Fig 2E; S1–S3 Figs) of the spatial working memory test in Tg mice. In both tasks, the early retardation shown in Tg mice was attenuated as the mice performed more learning trials. These results were not due to overall motor deficit or alterations in emotional states because there were no differences between WT and Tg mice in the open field test. The abnormal regulation of synaptic plasticity shown in the physiological experiment, particularly the impairment of early NMDAR-dependent LTP (Fig 6B and 6C), could provide a clue to the mechanisms underpinning the transient behavioral deficit we observed. A causal relationship between enhanced hippocampal LTP and memory deficit has been provided by the disruption of genes encoding the synaptic scaffold PSD-95 [31] or ionotropic GluR2 subunit [32] or by a recently developed mouse model of Alzheimer’s disease [33]. However, impaired DHPG-LTD did not appear to interfere with normal retention performance during the late phase of learning, consistent with previous studies showing a relatively minor influence of a LTD deficit on hippocampal-dependent learning, such as that examined using the water maze [34–36]. Therefore, the transient working memory deficit found in Tg mice could be caused in part by altered LTP and LTD within related neuronal circuitry, including in the hippocampus.

### Localization of IP_3_K-A in presynaptic terminals and enhancement of basal presynaptic release and vesicle pool size following IP_3_K-A overexpression

Schaffer collateral-CA1 synapses in Tg mice showed a significant decrease in PPF reflecting an increased presynaptic release probability (Fig 3B). The biochemical data in the present study revealed that IP_3_K-A was detected in synaptic fractions, including crude synaptosomes, synaptic membranes, and synaptic vesicle fractions, of the mouse hippocampus, providing strong evidence for the presynaptic presence of IP_3_K-A. Therefore, we looked at the size of the presynaptic vesicle pools to determine whether a change there underpinned change in the presynaptic release probability. The results showed that the number of synaptic vesicles in the hippocampal CA3 terminals of Tg mice was significantly increased compared with that of the control mice, as assessed by EM analysis (Fig 5B and 5C). This is consistent with a previous immuno-EM ultrastructural study showing that IP_3_K-A is localized at axon terminals and associated with synaptic vesicles [15]. However, the mechanism whereby overexpressed IP_3_K-A modulates the vesicle pool size remains unknown.

Previous studies also showed that IP_3_K-A immunoreactivity is associated with the spine apparatuses and plasmalemma of pyramidal neurons in the cortex and hippocampus and is located in the dendritic spines of Purkinje and basket cells in the cerebellum [15], suggesting a postsynaptic function of IP_3_K-A. Our results showed that IP3K-A overexpression did not affect the spine density of mature neurons in adult mice, consistent with a previous study that found that overexpression or deletion of IP_3_K-A has few effects on synaptic formation, spine density, and synaptic contacts of mature neurons [8]. However, in immature neurons, overexpression of IP_3_K-A increases the number of dendritic protrusions [8]. Although the postsynaptic function of IP_3_K-A is still unclear, IP_3_K-A may play a role in the trafficking of AMPAR-containing postsynaptic vesicles.

### PKC role is changed by IP_3_K-A overexpression during both basal presynaptic release and DHPG-LTD

Stimulation of mGluR activates PLC, leading to the production of DAG and IP_3_ from PIP_2_. The PKC enzymes are activated by intracellular signals such as increased DAG [22] or Ca^2+^ [23]. Regarding the role of PKC activity, our results showed that the basal presynaptic release probability is reduced by PKC activity in WT mice but is enhanced in Tg mice. Furthermore, DHPG-LTD is expressed PKC-independently in WT mice, consistent with previous studies [24–30]. PKC activation after PLC-induced DAG and IP_3_ signaling appears to not be essential for the formation of stable DHPG-LTD in WT mice. However, DHPG-LTD is PKC-dependent in Tg mice, in which basal PKC activity may be increased. Overexpression of IP_3_K-A changed the role of PKC activity at both presynaptic sites, such that activated PKC increased presynaptic intracellular Ca^2+^ levels via an unknown mechanism and was involved in the decay of the postsynaptic contribution to DHPG-LTD.

DAG signaling by diacylglycerol kinase iota (DGKι) has been recently implicated in mGluR-dependent LTD, suggesting that DGKι-induced DAG elimination promotes the reduction of transmitter release via inhibiting PKC activity [37]. However, there are studies showing that PLC and PKC can work separately in diverse signaling conditions. In spinal sensory synapses, DHPG-LTD was prevented by preincubation with thapsigargin, cyclopiazonic acid, or 2-APB, but not by ryanodine, a Ca^2+^-induced Ca^2+^ release inhibitor, and PKC inhibitors [28], which is consistent with our results. In basolateral amygdala synapses, DHPG-LTD was induced via a PLC-dependent but PKC-independent mechanism, whereas the priming action of mGluR5Rs on basolateral amygdala LTP was both PLC- and PKC-dependent. It has also been shown that presynaptic muscarinic M1 receptor activation is necessary for PKC activation during DHPG-LTD [38]. These results, including ours, suggest that PKC roles change dynamically depending on the stimulating input pathways during synaptic transmission, and plasticity, such as during DHPG-LTD.

### IP_3_R signaling role in presynaptic release process is unaltered in Tg mice

Neurotransmitter release from presynaptic terminals is altered by intracellular Ca^2+^ levels, which are regulated by the influx of extracellular Ca^2+^ ions and the release and uptake of intracellular Ca^2+^ stores [39, 40]. The Ca^2+^ release from the ER is mediated by ryanodine receptors and IP_3_Rs, which are gated by intracellular Ca^2+^ and IP_3_, respectively [41]. Thus, IP_3_K-A might be associated with Ca^2+^ efflux from the ER. However, our results with 2-APB, IP_3_R antagonist, showed that IP_3_R signaling was negatively involved in presynaptic release in both WT and Tg mice and that IP_3_K-A overexpression did not significantly change the role of IP_3_R in this process.

When the mGluR signaling pathway is activated by DHPG, the amount of IP_3_ is expected to increase transiently due to the subsequent IP_3_K-A activity. Our results showed that the presynaptic release probability was mildly increased during DHPG-LTD in WT mice (Fig 7A). When IP_3_R was inhibited, the increased presynaptic release probability was much greater during DHPG-LTD than it was during basal synaptic transmission (Fig 7D, middle plot, and Fig 7E). In addition, either IP_3_K-A overexpression or IP_3_R inhibition increased the presynaptic release probability during DHPG-LTD. It is expected that IP_3_K-A overexpression would deplete IP_3_, resulting in IP_3_R inhibition. However, it is unknown how inhibition of IP_3_R leads to the observed increase in presynaptic release probability. One possibility is that the increased IP_4_ level, following IP_3_K-A-mediated conversion of IP_3_, may augment Ca^2+^ influx from the extracellular fluid through activation of voltage-gated Ca^2+^ channels [42], providing better conditions for presynaptic release when IP_3_R is inhibited by an antagonist or IP_3_ depletion. Another possibility is that overexpression of IP_3_K-A may lead to changes in the activity of proteins that modulate intracellular Ca^2+^ levels or in the metabolism of inositol polyphosphates, both of which would increase release probability. In IP_3_K-A KO mice, synaptosomes display a shortened duration of both IP_3_ signals and IP_3_-dependent Ca^2+^ transients due to the compensatory up-regulation of IP_3_ phosphatase and the ER Ca^2+^ ATPase pump 2b proteins, which reduce intracellular Ca^2+^ levels [8]. If these compensatory regulations work in the opposite direction in IP_3_K-A-overexpressing neurons, a transient increase in intracellular Ca^2+^ levels may increase the release probability of Tg neurons.

The roles of postsynaptic IP_3_Rs in synaptic plasticity are quite complex and vary depending on the types of neurons. First, IP_3_Rs play a role in the Ca^2+^ increase in dendrites and by somatic and nuclear Ca^2+^ signals [43]. Second, both the GluA2 exit from the ER and the synaptic insertion, and the synaptic depression by the concomitant synaptic GluA1 endocytosis, depend on IP_3_R- and ryanodine receptor-controlled Ca^2+^ release in medium spiny neuron cultures [44, 45] and in the suppression of AMPAR currents by orexin-A in retinal ganglion cells [46]. Third, postsynaptic IP_3_R is also involved in AMPAR insertion during spike timing-dependent plasticity [47] and in both muscarinic and cholinergic enhancement of AMPAR-mediated transmission in CA1 pyramidal neurons [48, 49]. The mechanisms underlying the roles of postsynaptic IR_3_Rs remain to be determined in the DHPG-LTD model, especially in Tg mice.

### Differential roles of overexpressed IP_3_K-A during DHPG-LTD and NMDAR-dependent LTP

Increases in presynaptic release probability and postsynaptic Ca^2+^ release from stores have been suggested to explain early LTP expression [50]. Our results examining LTP in WT mice could be explained by an enhanced Ca^2+^-dependent presynaptic neurotransmission as shown by changes in PPF (Fig 6B and 6C). However, our results in Tg mice suggested both postsynaptic and presynaptic contributions to LTP. In single-theta-burst LTP experiments, early LTP in Tg mice was greater than that in WT mice, while presynaptic PPF changes were similar, indicating an additional postsynaptic role in Tg mice (Fig 6B). Results with four-theta-burst LTP further indicated that early LTP was primarily expressed postsynaptically in Tg mice because the PPF reduction was much less in Tg mice than in WT mice (Fig 6C). Therefore, these results with Tg mice revealed an additional postsynaptic role of overexpressed IP_3_K-A for the enhancement of early LTP. Because postsynaptic IP_4_, but not IP_3_, can increase LTP [42], it is possible that a mechanism involving IP_4_ might enhance early LTP in Tg mice.

An analysis of LTD with the PPF change also provided a clue to the source of LTD expression. In WT mice, induction of LTD appeared immediately after DHPG washout, and PPF was reduced during most of the DHPG-LTD (Fig 7A, by approximately 7% at 60–70 min, *p* < 0.0001). The PPF reduction should have caused an increase in the fEPSP, but LTD was still induced. This suggested that both presynaptic and postsynaptic components are involved in the expression of DHPG-LTD and that inhibitory postsynaptic processes can overcome the potential increase in fEPSP induced by presynaptic components, resulting in a net reduction in the postsynaptic AMPAR current for DHPG-LTD expression. Compared with WT mice, Tg mice displayed a reduction in DHPG-LTD (Fig 7A, upper panel) and PPF (Fig 7A, lower panel, and Fig 7F) in CA1 synapses of young mice, while NMDAR-dependent LTD was normal (Fig 6A). This finding in Tg mice indicated that LTD expression consists of different phases, with both presynaptic and postsynaptic contributions. Relative to that in WT mice, during the early phase of LTD in Tg mice, a larger, lasting PPF reduction (reduced approximately 19% at 70 min) appeared immediately, which might inhibit LTD expression via increased presynaptic release. However, early LTD still occurred, suggesting strong postsynaptic components for early LTD expression. Blockade of LTD developed slowly while PPF was reduced, suggesting a decay of the early postsynaptic contribution. These results indicated that overexpressed IP_3_K-A can increase synaptic function either presynaptically or postsynaptically with different temporal kinetics. In addition, the results of our PKC inhibitor experiments suggested a role of PKC in the decay of the early postsynaptic contribution to DHPG-LTD in Tg mice.

### Interaction of IP_3_K-A, IP_3_R, and PKC with one another

Reduced presynaptic release probability [51, 52] and postsynaptic AMPAR endocytosis have been suggested [44, 45, 51, 53] as mechanisms for DHPG-LTD expression. Our results showing that a molecule can play different roles depending on the phase of LTD add complexity to the understanding of the DHPG-LTD expression mechanism. Our results indicated that both IP3K-A-dependent presyntaptic and postsynaptic components are involved in the early phase of DHPG-LTD in WT mice, but the postsynaptic contribution is reduced in the late phase. The PKC inhibitors did not block DHPG-LTD in WT mice but blocked the effect of overexpressed IP_3_K-A in DHPG-LTD expression, which suggested that PKC is activated in the overexpressed IP_3_K-A signaling pathway. The effects of the IP_3_R antagonist differed from those of the PKC inhibitors. The IP_3_R antagonist blocked DHPG-LTD in both WT and Tg mice without blocking the reduction of PPF during DHPG-LTD. These results suggested that the PKC activity-dependent pathway and the IP_3_R-dependent pathway are differentially regulated by overexpressed IP_3_K-A during DHPG-LTD expression. Such a timely modulation has been shown in an *Aplysia* model system, where induction of long-term facilitation comprises the early short-term state, in which spontaneous release increases, and the intermediate-term stage, in which increases of postsynaptic IP_3_ and intracellular Ca^2+^ result in the recruitment of AMPAR clusters [54].

A summary of the DHPG-LTD model based on the results of the present study follows. In WT mice, the initial strength of the postsynaptic component is maintained constant, continuously overcoming the presynaptic component. In Tg mice, the strength of both the presynaptic and postsynaptic components is increased relative to that in WT mice. However, the much stronger postsynaptic component decays over a time, a process that is PKC-dependent. Thus, overexpressed IP_3_K-A appears to enhance both presynaptic and postsynaptic components as well as the late decay of the postsynaptic component in DHPG-LTD. Therefore, our results in Tg mice indicated that DHPG-LTD is expressed by a dynamic regulation of both presynaptic and postsynaptic function. Without these Tg mice, these dynamic regulatory roles of IP_3_K-A on synaptic function might not have been discovered.

In conclusion, overexpression of IP_3_K-A in the forebrain of the mouse increased the presynaptic vesicle pool of CA3 neuron terminals, presynaptic release probability, and basal postsynaptic AMPAR currents without significant effects on the spine density or morphology of CA1 neurons. The PKC-independent mGluR-LTD in WT mice was converted to PKC-dependent in mice overexpressing IP_3_K-A. The very early period of NMDAR-dependent LTP was selectively enhanced in Tg mice. The synaptic transmission of hippocampal CA1 neurons in WT mice appeared to be modulated by interactions among IP_3_K-A, PKC, and IP_3_R activities, which changed depending on the input signaling path. In IP_3_K-A overexpression, DHPG-LTD is impaired by mechanisms that are PKC- and IP_3_R-dependent. The present results using a gain of function approach together with those of previous KO studies show that abnormal expression of IP_3_K-A reveals unique and complex roles of IP_3_K-A in NMDAR- and mGluR-dependent synaptic plasticity models and some hippocampal-dependent learning tasks.

## Supporting information

S1 Fig IP_3_K-A overexpression at 4-week-old mice. IP_3_K-A overexpression was induced in the hippocampus of young mice by Dox administration through breast-feeding. Following electrophysiological analysis in the 4-week-old WT and Tg mice brain, CA1 and CA3 tissues were analyzed using Western blot.
(TIF)

S2 Fig Dose-dependent Dox-mediated IP_3_K-A expression in the hippocampus. Tg mice fed Dox at the indicated dose for 2 weeks. IP_3_K-A protein expression of brain sagittal section was detected with anti-IP_3_K-A and Cy3-conjugated secondary antibody.
(TIF)

S3 Fig Expression of Dox-dependent and reversible IP_3_K-A in Tg mice. Brain lysates were immunoblotted with IP_3_K-A and β-actin antibodies. The mice were fed Dox-containing food for 0, 3, 6, 9, 12, or 15 days, and after 15 days of feeding Dox-containing food, fed Dox-free food for 7, 14, 21, 28, 35, 42 or 49 days, respectively. Hippocampal lysates were prepared at each time point. Time-dependent expression of the IP_3_K-A protein was analyzed by immunoblotting with IP_3_K-A and β-actin antibodies (A) and quantified (B).
(TIF)

S4 Fig Total visits to arms in radial arm maze. Tg mice showed increased total visits in the radial arm maze test. Tg mice tended to show increased total visits in early sessions (S1-S3) (A) and showed a higher number of visits in early phase (S1-S3), but not late phase (S4-S7) (B) (WT, n = 15; Tg, n = 12).
(TIF)

S5 Fig Unchanged expression levels of synaptic proteins. Hippocampal lysates from adult WT or Tg mice were analyzed using Western blot with several antibodies of synaptic molecules (left). Densitometric quantification normalized with GAPDH did not show any significant difference between WT and Tg mice except for IP_3_K-A (right, n = 4).
(TIF)

S6 Fig Changes in basal synaptic properties caused by the application of chemical compounds used in Fig 7. (A1–A3), 1 μM Rö-31-8220 pre-incubation. (A1), fEPSP slope vs. presynaptic fiber volley amplitude plot. (A2), linear regression slope values of each slice from the fEPSP I-O plot (A1). WT, 2.34 ± 0.26, n = 12 slices; Tg, 2.25 ± 0.11, n = 15; *p* = 0.72. (A3), the PPF ratios at five intervals showed no significant difference between WT and Tg mice. (B1–B3), 1 μM Calphostin C pre-incubation. (B1), fEPSP slope vs. presynaptic fiver volley amplitude plot. (B2), linear regression slope values of each slice from the fEPSP I-O plot (left). WT, 2.05 ± 0.37, n = 6; Tg, 1.97 ± 0.16, n = 6; *p* = 0.84. (B3), the PPF ratios at five intervals showed no significant difference between WT and Tg mice. (C1–C3), 16 μM 2-APB pre-incubation. (C1), fEPSP slope vs. presynaptic fiver volley amplitude plot. (C2), linear regression slope values of each slice from the fEPSP I-O plot (C1). WT, 2.18 ± 0.17, n = 15; Tg, 2.45 ± 0.19, n = 20; *p* = 0.32. (C3), the PPF ratios at five intervals showed no significant difference between WT and Tg mice.
(TIF)

S7 Fig PP-LFS LTD on the synaptic strength of young CA1 synapse. Upper plot, PP-LFS produced LTD of young CA1 synapses of both WT and Tg mice. LTD was induced by PP-LFS protocol (1Hz, 900 paired-pulses at 40 msec interval) at the CA1 dendritic region of the hippocampal slice. The slope of fEPSP was 86.9 ± 1.4% (WT, n = 8 slices) and 83.3 ± 0.6% (Tg, n = 10) at 60–65 min (*grey bar*, *p* = 0.11). Lower plot shows the normalized PPF ratio of the fEPSP slopes and the average values at 60–65 min were statistically compared (WT, 1.04 ± 0.57E-2; Tg, 1.09 ± 0.55E-2, *p* < 0.0001).
(TIF)

S8 Fig Pre-incubation effects of 2 μM KN62 (or KN93) on DHPG-LTD. (A), Basal I-O plot in KN62 (or KN93) pre-incubated slices. Left, fEPSP slope vs. presynaptic fiver volley amplitude plot. Control (0.5% DMSO or 2 μM KN92), n = 23 slices; KN62 or KN93, n = 28. Right, linear regression slope values of each slice from the fEPSP I-O plot (left). WT, 2.18 ± 0.16; Tg, 2.25 ± 0.12, n = 15; *p* = 0.71. (B), Paired pule ratio in KN62 (or KN93) pre-incubated slices. PPF ratios at the five intervals indicated no significant difference between wild type and OX mutant mice. (C), DHPG-induced LTD in KN62 (or KN93) pre-incubated slices. 2HM KN62 (or KN93) or control 0.5% DMSO (or 2μM KN92) was applied during the whole recording period. 10 minute application of DHPG (50μM) caused LTD of CA1 synapses in wild type mice (measured at 51–60 min, control: 81.4 ± 0.4% of baseline, n = 23, *p* < 0.001, KN62/KN93: 79.4 ± 0.3%, n = 28, *p* < 0.001).
(TIF)
