# Supplementary figures and images for: Inositol 1,4,5-trisphosphate 3-kinase A overexpressed in mouse forebrain modulates synaptic transmission and mGluR-LTD of CA1 pyramidal neurons
Source: PLoS One. 2018 Apr 4;13(4):e0193859. doi: 10.1371/journal.pone.0193859 (PMC5884490; doi:10.1371/journal.pone.0193859)

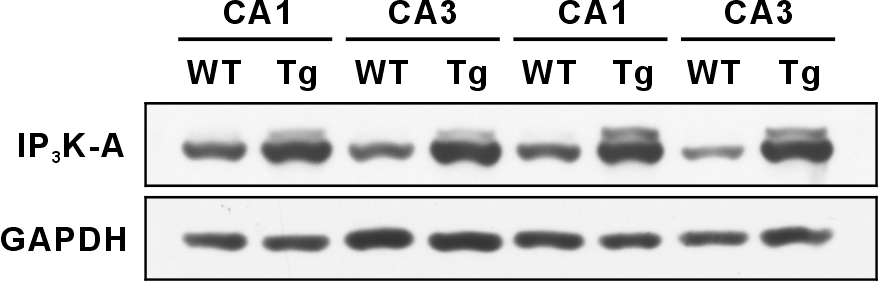

Supplement: S1 Fig — IP3K-A overexpression was induced in the hippocampus of young mice by Dox administration through breast-feeding. Following electrophysiological analysis in the 4-week-old WT and Tg mice brain, CA1 and CA3 tissues were analyzed using Western blot. (TIF) [file pone.0193859.s001.tif]

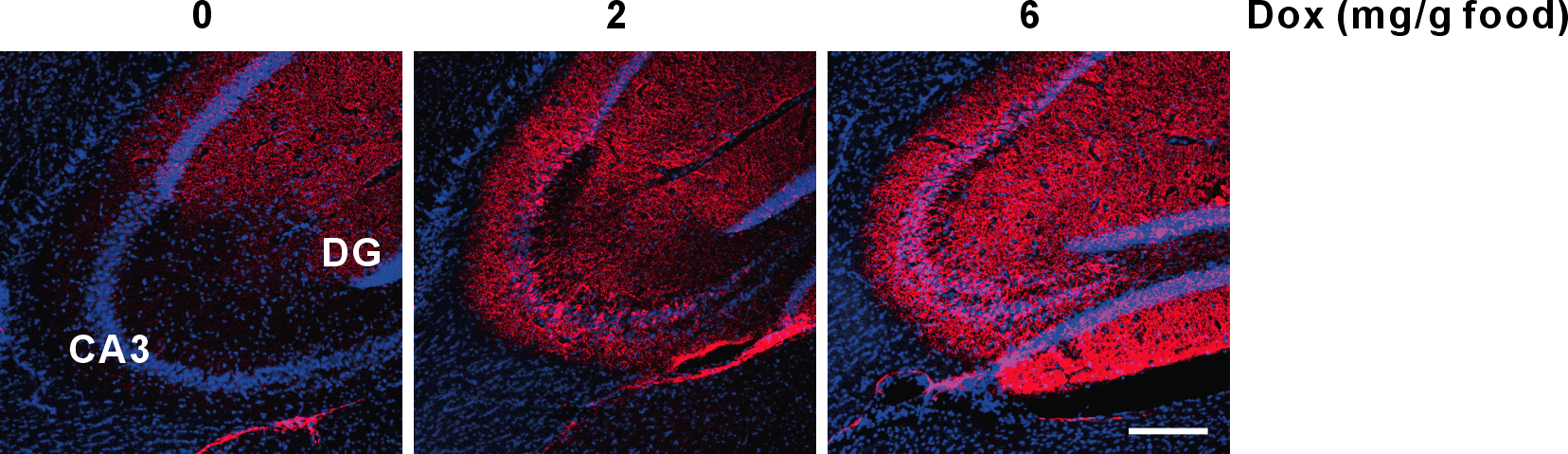

Supplement: S2 Fig — Tg mice fed Dox at the indicated dose for 2 weeks. IP3K-A protein expression of brain sagittal section was detected with anti-IP3K-A and Cy3-conjugated secondary antibody. (TIF) [file pone.0193859.s002.tif]

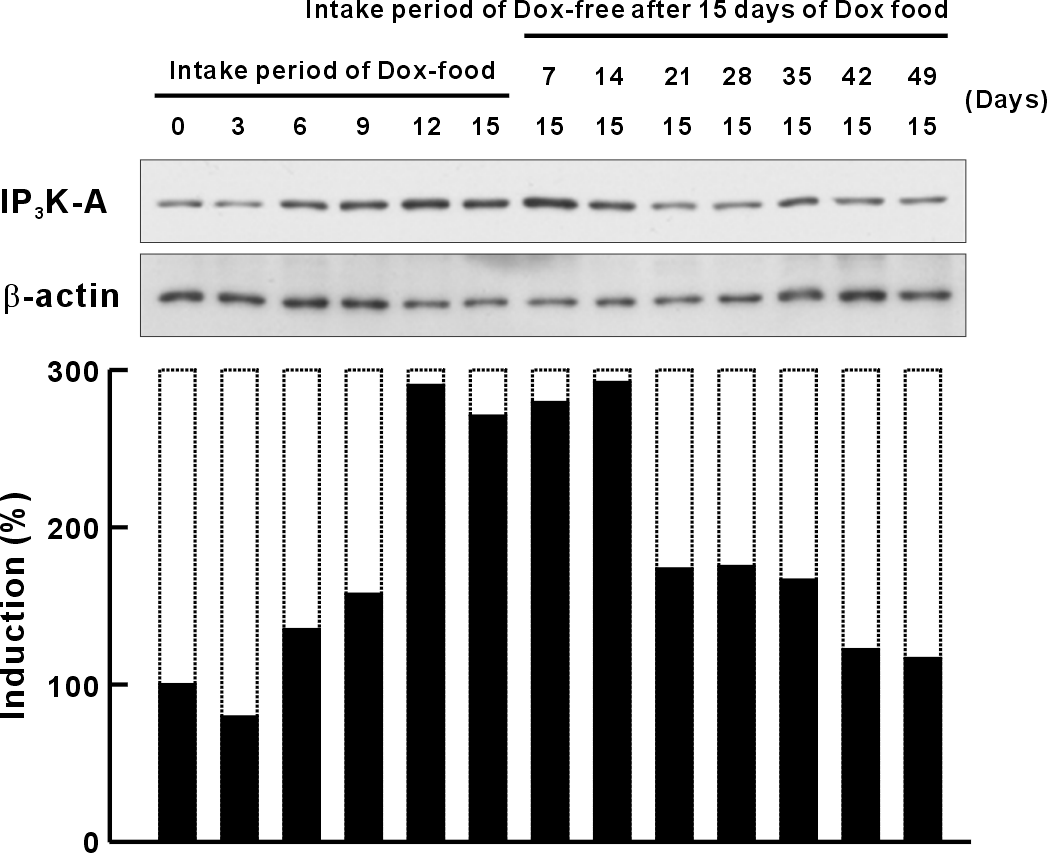

Supplement: S3 Fig — Brain lysates were immunoblotted with IP3K-A and β-actin antibodies. The mice were fed Dox-containing food for 0, 3, 6, 9, 12, or 15 days, and after 15 days of feeding Dox-containing food, fed Dox-free food for 7, 14, 21, 28, 35, 42 or 49 days, respectively. Hippocampal lysates were prepared at each time point. Time-dependent expression of the IP3K-A protein was analyzed by immunoblotting with IP3K-A and β-actin antibodies (A) and quantified (B). (TIF) [file pone.0193859.s003.tif]

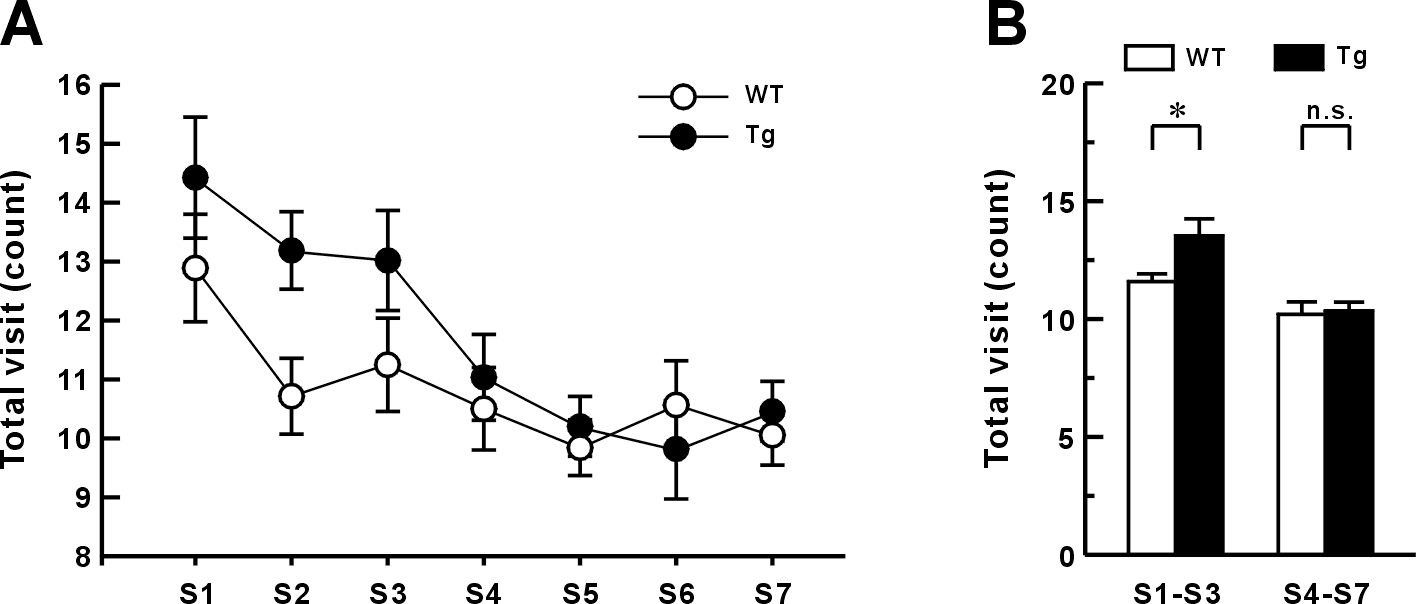

Supplement: S4 Fig — Tg mice showed increased total visits in the radial arm maze test. Tg mice tended to show increased total visits in early sessions (S1-S3) (A) and showed a higher number of visits in early phase (S1-S3), but not late phase (S4-S7) (B) (WT, n = 15; Tg, n = 12). (TIF) [file pone.0193859.s004.tif]

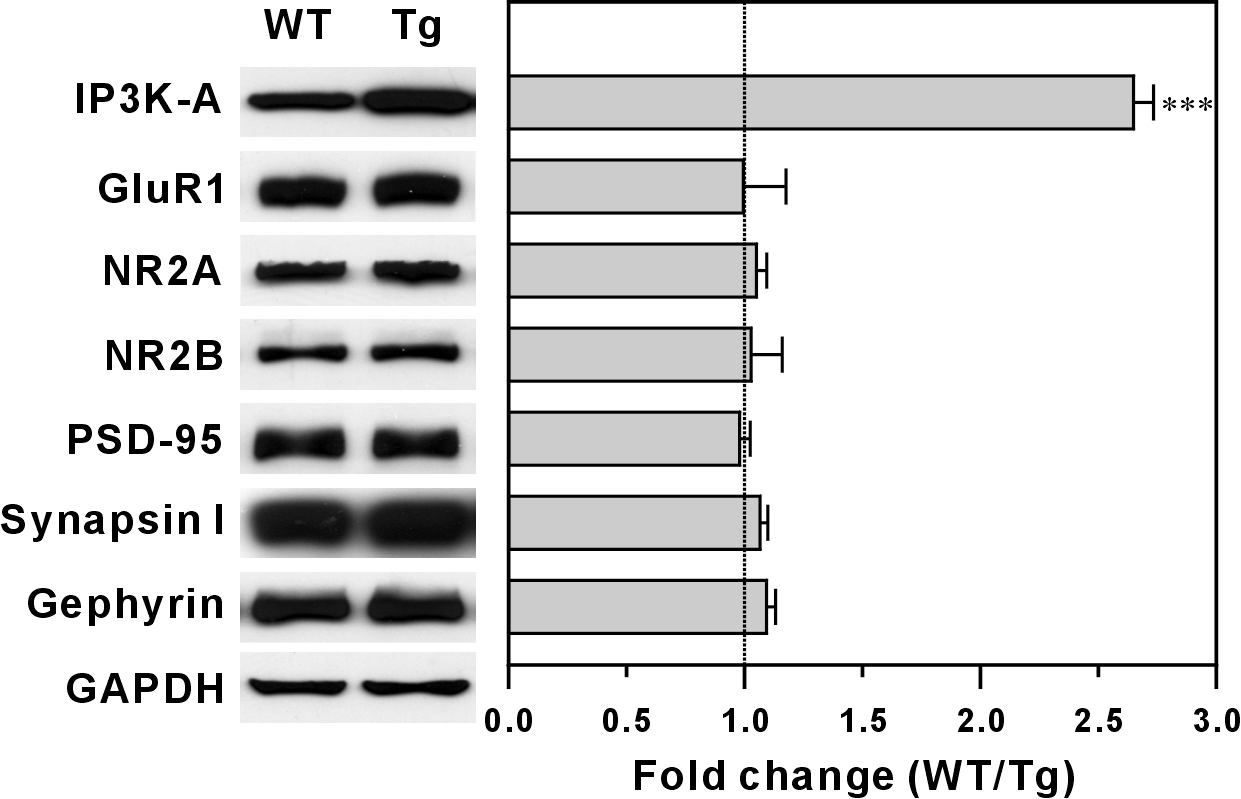

Supplement: S5 Fig — Hippocampal lysates from adult WT or Tg mice were analyzed using Western blot with several antibodies of synaptic molecules (left). Densitometric quantification normalized with GAPDH did not show any significant difference between WT and Tg mice except for IP3K-A (right, n = 4). (TIF) [file pone.0193859.s005.tif]

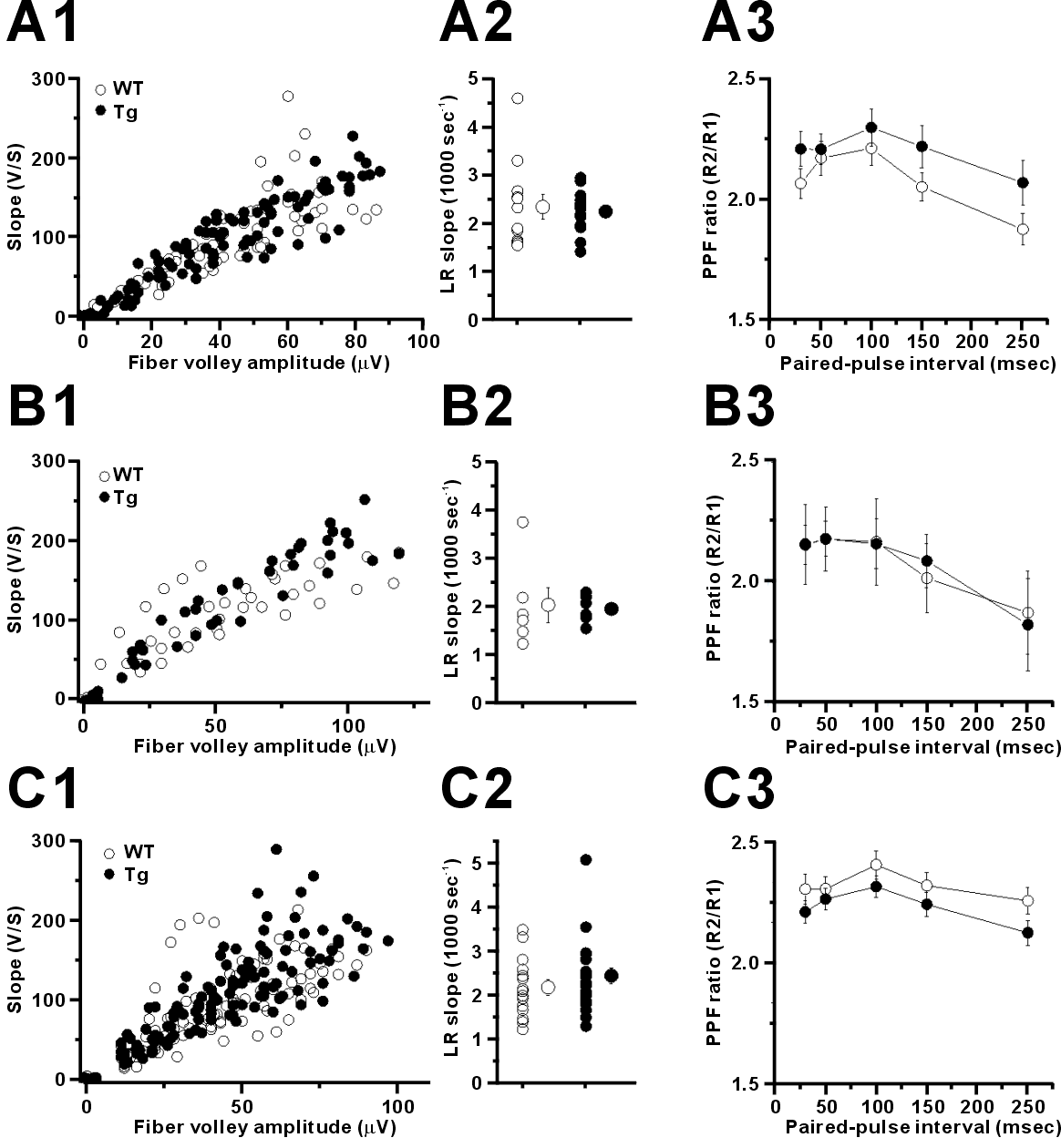

Supplement: S6 Fig — (A1–A3), 1 μM Rö-31-8220 pre-incubation. (A1), fEPSP slope vs. presynaptic fiber volley amplitude plot. (A2), linear regression slope values of each slice from the fEPSP I-O plot (A1). WT, 2.34 ± 0.26, n = 12 slices; Tg, 2.25 ± 0.11, n = 15; p = 0.72. (A3), the PPF ratios at five intervals showed no significant difference between WT and Tg mice. (B1–B3), 1 μM Calphostin C pre-incubation. (B1), fEPSP slope vs. presynaptic fiver volley amplitude plot. (B2), linear regression slope values of each slice from the fEPSP I-O plot (left). WT, 2.05 ± 0.37, n = 6; Tg, 1.97 ± 0.16, n = 6; p = 0.84. (B3), the PPF ratios at five intervals showed no significant difference between WT and Tg mice. (C1–C3), 16 μM 2-APB pre-incubation. (C1), fEPSP slope vs. presynaptic fiver volley amplitude plot. (C2), linear regression slope values of each slice from the fEPSP I-O plot (C1). WT, 2.18 ± 0.17, n = 15; Tg, 2.45 ± 0.19, n = 20; p = 0.32. (C3), the PPF ratios at five intervals showed no significant difference between WT and Tg mice. (TIF) [file pone.0193859.s006.tif]

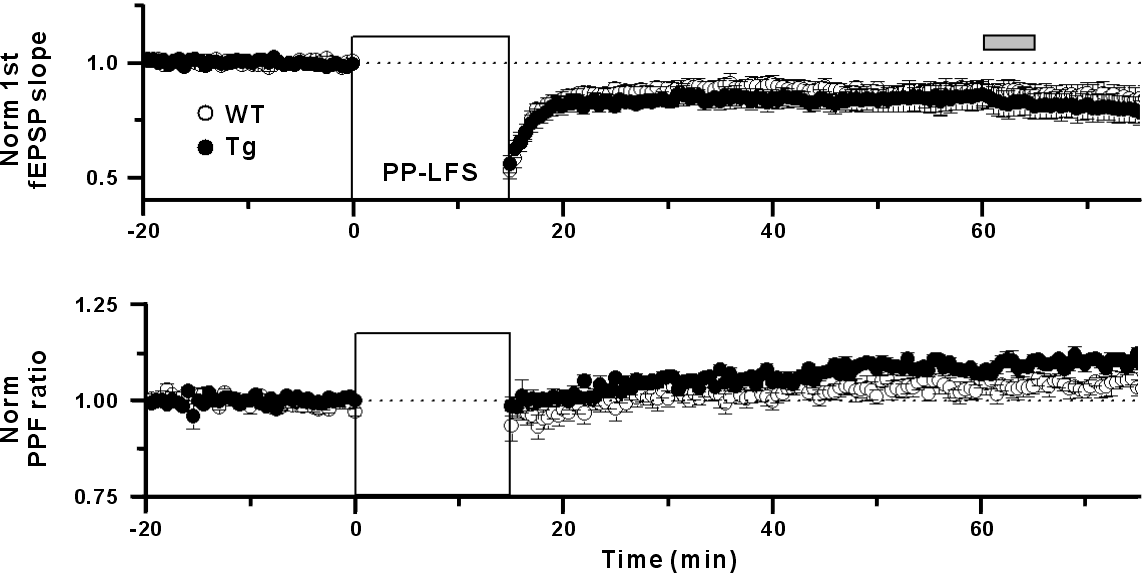

Supplement: S7 Fig — Upper plot, PP-LFS produced LTD of young CA1 synapses of both WT and Tg mice. LTD was induced by PP-LFS protocol (1Hz, 900 paired-pulses at 40 msec interval) at the CA1 dendritic region of the hippocampal slice. The slope of fEPSP was 86.9 ± 1.4% (WT, n = 8 slices) and 83.3 ± 0.6% (Tg, n = 10) at 60–65 min (grey bar, p = 0.11). Lower plot shows the normalized PPF ratio of the fEPSP slopes and the average values at 60–65 min were statistically compared (WT, 1.04 ± 0.57E-2; Tg, 1.09 ± 0.55E-2, p < 0.0001). (TIF) [file pone.0193859.s007.tif]

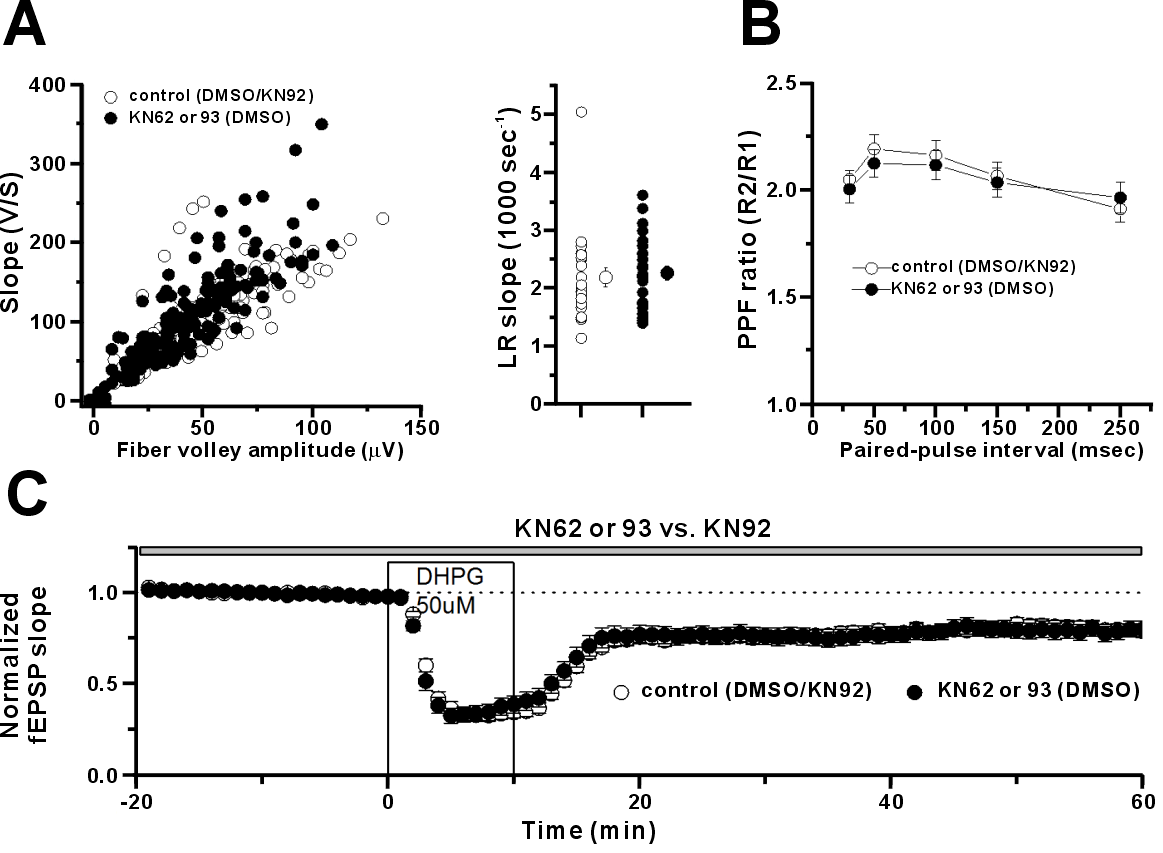

Supplement: S8 Fig — (A), Basal I-O plot in KN62 (or KN93) pre-incubated slices. Left, fEPSP slope vs. presynaptic fiver volley amplitude plot. Control (0.5% DMSO or 2 μM KN92), n = 23 slices; KN62 or KN93, n = 28. Right, linear regression slope values of each slice from the fEPSP I-O plot (left). WT, 2.18 ± 0.16; Tg, 2.25 ± 0.12, n = 15; p = 0.71. (B), Paired pule ratio in KN62 (or KN93) pre-incubated slices. PPF ratios at the five intervals indicated no significant difference between wild type and OX mutant mice. (C), DHPG-induced LTD in KN62 (or KN93) pre-incubated slices. 2HM KN62 (or KN93) or control 0.5% DMSO (or 2μM KN92) was applied during the whole recording period. 10 minute application of DHPG (50μM) caused LTD of CA1 synapses in wild type mice (measured at 51–60 min, control: 81.4 ± 0.4% of baseline, n = 23, p < 0.001, KN62/KN93: 79.4 ± 0.3%, n = 28, p < 0.001). (TIF) [file pone.0193859.s008.tif]
